# Network theory of the bacterial ribosome

**DOI:** 10.1371/journal.pone.0239700

**Published:** 2020-10-05

**Authors:** Laurie E. Calvet, Serhii Matviienko, Pierre Ducluzaux

**Affiliations:** CNRS, Centre de Nanosciences et Nanotechnologies, Université Paris-Saclay, Palaiseau, France; Ohio State University, UNITED STATES

## Abstract

In the past two decades, research into the biochemical, biophysical and structural properties of the ribosome have revealed many different steps of protein translation. Nevertheless, a complete understanding of how they lead to a rapid and accurate protein synthesis still remains a challenge. Here we consider a coarse network analysis in the bacterial ribosome formed by the connectivity between ribosomal (r) proteins and RNAs at different stages in the elongation cycle. The ribosomal networks are found to be dis-assortative and small world, implying that the structure allows for an efficient exchange of information between distant locations. An analysis of centrality shows that the second and fifth domains of 23S rRNA are the most important elements in all of the networks. Ribosomal protein hubs connect to much fewer nodes but are shown to provide important connectivity within the network (high closeness centrality). A modularity analysis reveals some of the different functional communities, indicating some known and some new possible communication pathways Our mathematical results confirm important communication pathways that have been discussed in previous research, thus verifying the use of this technique for representing the ribosome, and also reveal new insights into the collective function of ribosomal elements.

## Introduction

The wealth of high resolution images of the ribosome have revealed many of the steps involved in its function [1–4]. Throughout this vast literature, the importance of connectivity has often been remarked and discussed, however, only a few papers have considered viewing the connections as a network. Some explorations considered using the nucleotides or amino acids as nodes and their interactions as edges [5, 6], and showed that a network analysis highlights important mutations that have deleterious effects on ribosome function or highlighted highly conserved residues, drug binding sites and/or allosteric pathways. Other investigations considered interactions involving just ribosomal (r)proteins within each subunit [2, 7] and revealed that the interactions are highly conserved [7]. Here we consider a network analysis based on the interactions between RNAs and ribosomal (r)proteins [8]. The motivation for this research is to explore mathematically how ribosomal elements function together and determine important communication pathways.

While a large body of research exploring functionality provides significant insights into individual or a few ribosomal elements, collective interactions that allow the ribosome to achieve a particular function are still difficult to understand. In analogy to understanding the brain connectome [9], which provides new insights into functionality of sub-circuits, exploring the topological structure of the connectivity in the ribosome should provide analogous insights. The elongation cycle during protein synthesis can be broadly grouped into: i) decoding, where the tRNA is recognized to match the mRNA codon, ii) the formation of the peptide bond between the aminoacyl-tRNA and the nascent protein chain and iii) translocation where the ribosome moves along the mRNA in preparation for a new tRNA. We analyze the networks of *Thermus thermophilus* in four different steps of protein synthesis and also compare it with *E*. *Coli* in the decoding step. We use the high resolution structural files deposited in the protein databank [10]. To provide snapshots of the ribosome at the A/T or pre-accommodation state in the closed configuration after successful decoding we use pdb files 4v5g (*Thermus Thermophilus)* [11] and 5we4 (*E*. *coli*) [12]. This state contains the ternary complex (TC), which includes the elongation factor (EF)-TU, aminoacyl-tRNA and GTP. The second state is just after accommodation of the aminoacyl-tRNA onto the tRNA-A site after the EF-TU has been released, with tRNAs present on the A, P and E sites, pdb accession code 4y4p (*T*. *Thermophilus)* [13]. The next state (4v9h) we consider is an intermediate during translocation, known as the hybrid state, where the TRNAs have moved with respect to the large subunit but not the small subunit [14]. Finally the fourth state explored is just after translocation with the elongation factor (EF)-G still bound to the ribosome (pdb accession code: 4v5f) [15] but with tRNAs present only on the P and E sites.

**Fig 1:**
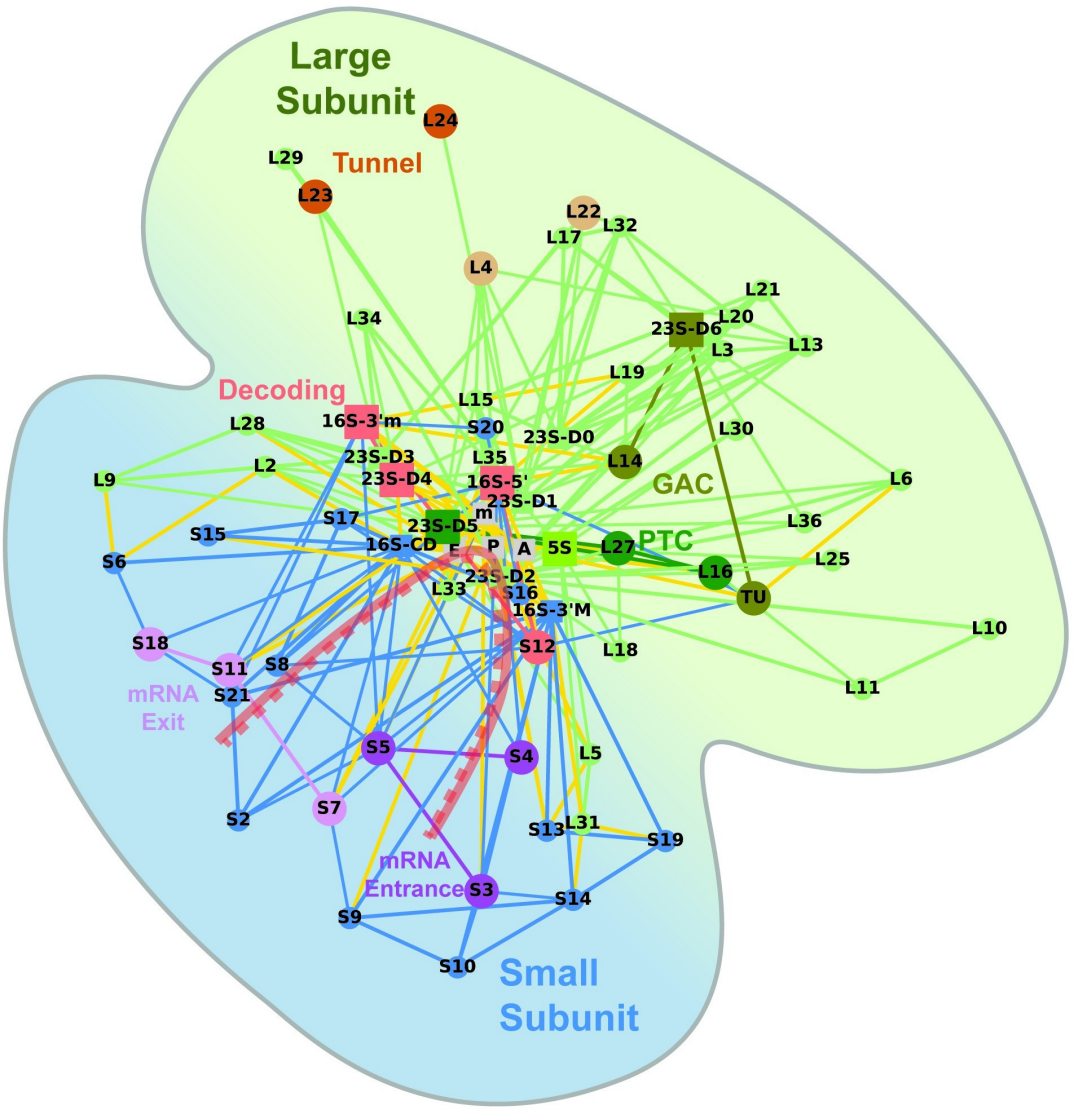
*E*. *coli* network (pdb accesion code 5we4) in the pre-accumulation (A/T) decoding step. This is an example of the interaction network in two dimenions, colored with respect to the large (green) and small (blue) subunits and where the different elements in the ribosome are placed according to a two-dimensional projection of their center of mass. In the large subunit three funtional groups are highlighted: 1) 23S-D5, L27 and L16 involved in formation of the peptide bond (dark green), 2) 23S-D6, L14, Elongation Factor TU concern the binding of GTPase (khaki) associated binding center (GAC), and 3) L4 and L22 (light brown) form a constriction near the mid-section of the peptide exit tunnel and rproteins L23, L24, (darker brown), as well as 23S-D3 and -D1 form tunnel the wall near the ribosomal surface. Also indicated is 5S rRNA (light green), which is thought to act as a transducer of information. In the small subunit, three highlighted functional groups are: 1) the decoding center (pink) involving 16S- 5’ and 16S-3’m, 23S-D4 and S12, 2) the mRNA entrance tunnel (S3, S4, S5 in dark purple) an 3) the mRNA exit tunnel (S7, S18, S11, in light purple). Intersubunit connections are depicted in yellow.

Fig 1 shows an example of the interaction network in two-dimensions, colored with respect to the large and small subunits and where the different elements in the ribosome are placed according to a two-dimensional projection of their center of mass. The colored nodes indicate some of the known functionalities of the different elements [16–20]. Nucleotides A1492 and A1493 of the 3’ minor domain (16S-3’m), G530 of the 5’ domain of 16S rRNA (16S-5’) and A1913 (domain 4) of 23S rRNA (23S-D4) allow for decoding of the tRNA [21]. 16S rRNA has also been shown to play an important role in initiation [22] via the anti-Shine-Dalgano sequence located in the 3’ minor domain (16S-3’m), and accuracy (16S-3’m). 5S rRNA is thought to act as a *physical transducer of information* [23] by providing connectivity between different functional centers. 23S plays an important role in the catalysis of the peptide bond (domain 5) [20], the GTP associated center (domain 6) and the formation of the tunnel [18].

The inherent multi-functionality of the rproteins, involving both biogenesis [16, 24] and protein synthesis [17], has made a thorough understanding their roles elusive [16, 17, 22, 23]. Proteins can be probed by knock-out, but such mutants are often not viable because of their essential role in assembly [25]. Other important techniques explore the effects of antibiotics [19, 26] and/or the creation of mutant rproteins with specific residues removed [27–29]. Explorations of allostery [30–32], ribosome heterogeneity and specialization [33, 34] have provided important new insights into communication and signaling. Since many interactions are determined during ribosome biogenesis, it is also likely that their role in this process is related to functionality during translation.

Ribosomal protein S12 is also thought to play an important role in decoding by aiding the formation of the conformational states adopted by the elongation factor EF-G [26]. The sarcin-ricin loop in domain 6 of the 23S rRNA and ribosomal proteins L14, L10, L11, L12 (note that the latter three are not present in the pdb file depicted in Fig 1), located in the stalk region (central protuberance), are involved with the GTPase Associated binding Center (GAC) [17, 35, 36]. L27 and L16 have been shown to play an important role [35] in peptide catalysis. The peptide exit tunnel can broadly be categorized into 3 regions: (a) the region near the peptidyl transferase center (PTC) dominated by 23S rRNA-domain 5, (b) a mid-region where L4 and L22 form a constriction and (c) a region near the ribosomal surface formed by 23S-domain 1, 23S-domain 3 and ribosomal proteins L23 and L24 (9,37). Protein L1 is thought to aid the release of the deacylated tRNA from the E-site [37]. Protein S3, S4 and S5 play a role in the formation of the mRNA entrance tunnel [38–40] and S4, S5 and S12 mutants were found to affect fidelity [19, 41]. S13 forms a link between the tRNA-P site and the head of the small subunit and modifications resulting in defects of subunit association have been shown to have an important impact on translation fidelity [42]. This protein, as well as S9 are known to play a role in proofreading [27, 42]. S7, S11 and S18 form the mRNA exit tunnel and may be involved in translation fidelity by providing structural support [40]. Finally, numerous elements have been identified as forming bridges between the small and large subunits [43]. The functionalities of the rproteins and rRNAs are summarized in Table 1.

**Table 1:** Summary of known functionalities of different elements in the ribosome.

Element	Function	References
16S-5', 3'm, 23S-D4, S12	Decoding	[21, 26]
16S-3'm	Initiation	[22]
16S-3'm, S14, S15, S12, S13, S9	accuracy of decoding	[19, 22, 27, 41, 42]
5S	transducer of information	[23]
23S-D5, L27, L16	PTC	[9, 18, 20, 37, 38]
23S-D6, L14, L10, L11, L12	GAC	[9, 17, 18, 35–37]
23S-D0, D5, L3	early section of tunnel	[46]
L4, L22	mid section of tunnel	[9, 37]
L23, L24, 23S-D3, D1	tunnel near surface	[9, 37]
S3, S4, S5	mRNA entrance tunnel	[38–40]
S7, S8, S11	mRNA exit tunnel and fidelity	[40]
L1	release of tRNA from E site	[37]
16S-3m, 16S-CD, 16S-5, 23S-D2, 23S-D4, 23S-D5, L2, L5, L14, L31, L19, S7, S13, S14, S15, S19	inter-subunit bridges	[43]

The network theory used in our analysis is an interdisciplinary methodology where the basic principles of graph theory are applied and extended to provide insights into the functionality and structure of different real world systems [8]. An important branch of network analysis has explored protein-protein interactions (PPIs) in the cell [44], which are much larger and more dynamic that the networks in the ribosome, and are known to regulate a wide array of biological processes. The goal of the analysis of PPIs is to understand the function of specific proteins, modular groups and the network as a whole through its structure. The important difference between PPI networks and those described here is the significant role in the connectivity of the RNAs. The 16S and 23S rRNAs are much larger molecules than the other elements in ribosome and to account for this we have considered the connectivity of their individual domains.

Real networks that are involved in the flow or the transfer of some particular element (for instance information, water, electricity, gas or people) are often described as small world [45]. Typical characteristics are high clustering, which enables mobilization of the element, and small distances between a given node and any other (average path length) to enable efficient transfer. Small world networks are like random graphs in that they have small path lengths but different in that they have much larger clustering coefficients. They can also be distinguished from regular networks like crystals that have large clustering coefficients but also have large average path lengths. The structure of random and regular networks is not particularly efficient for transferring information without an additional feature. For instance, in conducting crystals overlapping electronic wavefunctions can allow electrons to freely travel in the crystal creating an electron gas that is characterized by an efficient flow of charged carriers. Our first objective of the network analysis is to demonstrate the small world nature of the ribosomal networks to show that network theory can be useful for understanding the functionality of ribosomal elements.

We next focus on a centrality analysis, which determines the important hubs or communication centers. We explore degree, eigenvector, betweenness and closeness centralities. Our results reveal the dominance of rRNA domains in centrality measures concerned with the number of connections (degree and eigenvector) and show that rprotein hubs increase in importance in centrality measures concerned with the shortest distance between nodes (betweenness and closeness centralities). This result implies that rproteins are important potential mediators of information and should stimulate further detailed research that could enhance our understanding of the ribosome.

Finally, we explore the communities formed using modularity maximization [8], where nodes that have similar number of connections are grouped together. We find that the first decomposition reveals a few groups containing many elements that can be easily identified as functional units. Subsequent decompositions reveal smaller groups that depend strongly on the particular translational state. Our analysis reveals that some ribosomal elements remain in the same sub-network throughout elongation and others vary. While the functional groups are identified by the presence of certain elements which are therefore always members of the same group, other elements, which are also always associated with the group, provide new insights into their role during elongation. Likewise, the elements that change groups during elongation are likely to provide important communication pathways and/or multi-functionality.

## Materials and methods

To determine the interactions, we first isolate two individual elements in the ribosome. rRNAs, which are much longer molecules than the rproteins and make many more connections, are further divided into domains [46–48]. S1 Table shows the numbering of the nucleotides corresponding to the domains. Pymol [49] is then used to calculate the solvent accessible area of the pair (radius = 1.4 Å) and each individual to determine whether an interaction is present. This method of determining the interactions is the same method used for the automatic detection of rprotein interactions in ref [7]. We ignore multiple connections in order to focus on the functionality of the network. Larger values of the radius could be used to determine additional weaker interactions such as hydrogen bonds or van der Waals interactions.

A discussion of the nature of the extensions involved in many rprotein interactions was reported in [7] and it was noted that the majority of interfaces are phylogenetically conserved throughout evolution. The nature of the protein extensions, however, is neglected throughout this paper. Our analysis generated the list of interactions, reported in S2 Table. Elements of the biological ribosomal network in a given state may be missing for experimental reasons such as insufficient resolution, poorly resolved electron density maps or absence of proteins (specifically L1, L10, L11, L12, L9). Errors in the interactions may also occur due to poor resolution. Further, the ribosome is in a constant state of motion and therefore some contacts may be broken/formed with this motion resulting in interactions appearing in some files and not others.

An important subgroup of interactions involves connections between the small subunit and the large subunit. To compare our results with previously explored intersubunit bridges, we prepared S3 Table, which summarizes all known inter-subunit bridges in the bacterial ribosome [43] using our notations of rRNA domains. Our results found all of the previously known bridges as well as new inter-subunit interactions, which are detailed in S4 Table.

The first few rows of Table 2 report some of the characteristics of the networks considered here. The change in the size of the network can be related to the presence/absence of elongation factors and tRNAs or rproteins due to the different experimental conditions (notably L1, L9, L10, L12).

**Table 2:** Basic graph properties of the different pdb files considered in this paper.

pdb code	5we4	4v5g	4y4p	4v9h	4v5f
**ref**	12	11	13	14	15
**state**	pre-accommodation	pre-accommodation	accommodation	hybrid	post-elongation
**species**	*E*. *Coli*	*Thermophilus*	*Thermophilus*	*Thermophilus*	*Thermophilus*
**Method**	cryo-EM	X-ray	X-ray	X-ray	X-ray
**Resolution (Å)**	3.1	3.6	2.5	2.86	3.6
**Rms deviations:**					
**Bond length (Å)**	0.066	0.008	0.006	0.0004	0.008
**Bond angles (°)**	0.96	1.2	1.151	1.488	1.2
**Size**	68	68	65	68	67
**rRNA-rRNA**	5	8	9	8	6
**rprot-rprot**	57	52	55	60	59
**rRNA-rprot**	132	134	138	138	138
**mRNA and tRNA**	41	44	43	30	16
**# inter-subunit interactions**	18	19	19	16	18
**Order**	235	238	245	236	219
**Isolated**	0	0	0	0	0
**Pendant**	1	0	1	0	1
**Ave degree**	6.912	7.000	7.538	6.941	6.537
**Max degree**	23	26	24	26	25
**Diameter**	5	5	5	5	5
**Largest connected component**	1	1	1	1	1
**Ave path length**	2.540	2.480	2.440	2.480	2.550
**Cluster coefficient**	0.438	0.477	0.447	0.449	0.456
**Density**	0.103	0.104	0.118	0.104	0.099
**Assortativity**	-0.304	-0.279	-0.272	-0.262	-0.285

The table shows that the interactions are dominated by rRNA-rprotein connections. We observed many more inter-subunit interactions than previously reported. The small average path length and the high cluster coefficients are indicative of small world properties. Finally, the networks are found to be dis-assortative. A description of the corresponding calculation used to obtain the parameters in this table are provided in the methods section.

**Fig 2:**
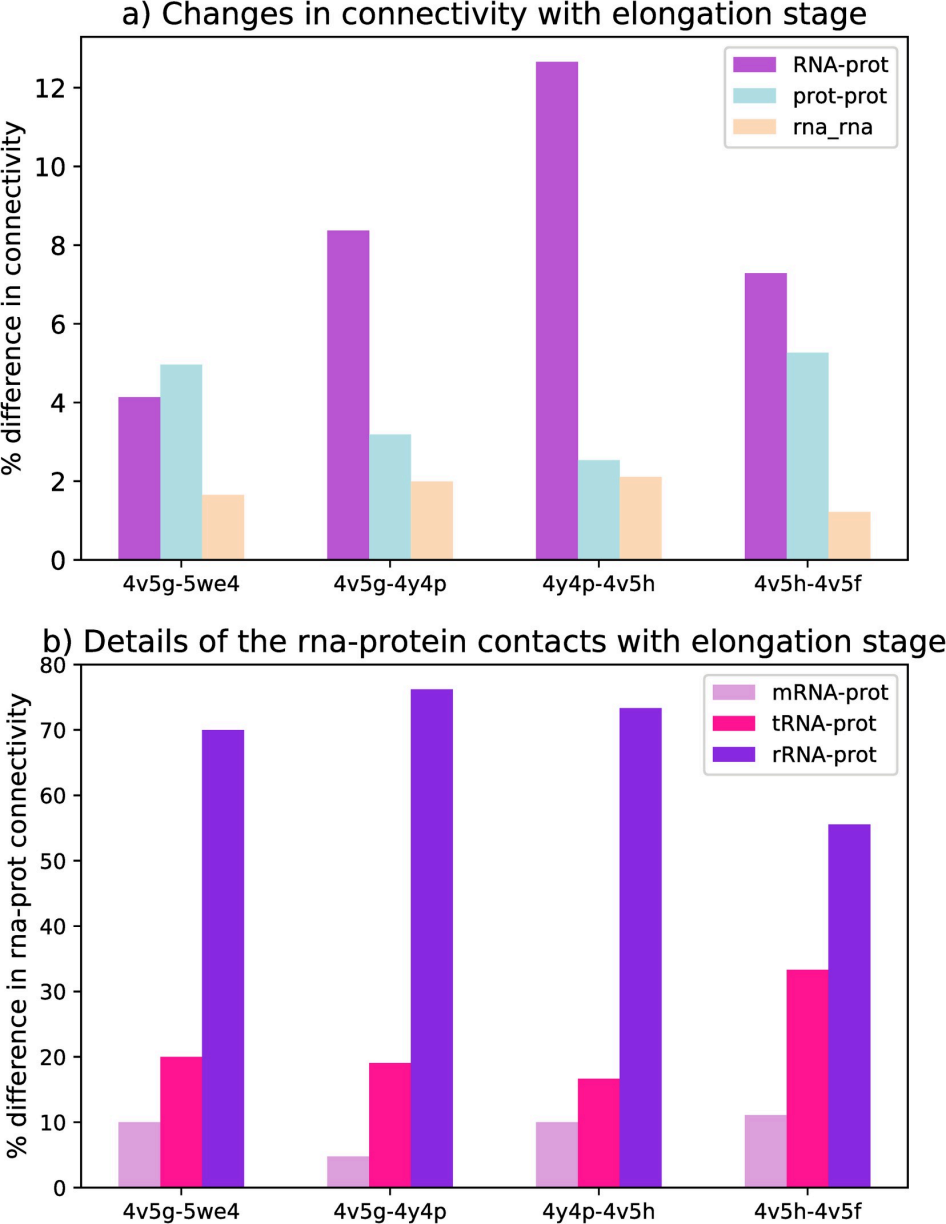
Percent difference in the connectivity. a) % difference in connectivity of the three principle interactions: RNA-protein, protein-protein and RNA-RNA. Note that the RNA refers to rRNA, mRNA and tRNA and the proteins refer to both ribosomal proteins and elongation factors. Only connections that are present in both files are included in the numerator but the denominator includes all interactions. b) % difference in the different RNA-protein connections, detailing the importance of the different RNAs. Note that 4v5g (*T*. *Thermophilus*) and 5we4 (*E*. *Coli*) are in the pre-accommodated state, 4y4p is in the accommodated state, 4v5h is in the hybrid state and 4v5f is the post-elongation state.

A comparison of changes in RNA-RNA, RNA-rprotein and rprotein-rprotein interactions in the different files is shown in Fig 2, where we have only included changes involving elements that are present in both pdb files. For instance, L1 is not present in the accommodated state (4y4p) but without a doubt it would still connect to tRNA-E in a functioning ribosome in the accommodated state, therefore its interactions are not included in the analysis in Fig 2. Another example is the connectivity of tRNA-A in the A/T state (pdb 4v5g) versus the accommodated state (4y4p) where the connections between tRNA-A and the large subunit proteins L16, L27 are missing because of the positioning of the tRNAs but since all the elements are present these differences are included. The total number of interactions (the denominator of the % difference), however, includes all unique connections in the two files. For these reasons, the estimation in Fig 2 should therefore be considered as conservative approximations.

We defined networks by including all proteins, mRNA, tRNAs and the different domains of the rRNAs available in a given pdb file as nodes and the interactions between them as edges. NetworkX [50] was used to create the graphs and determine the graph properties, which are now described. We define *k*_*i*_ as the number of connections of node *i*, known as the *degree*, denote <*k*> as its average, and the *maximum degree* as the number of maximum connections. The *diameter* of the graph is defined as the maximum number of nodes that must be traversed to connect the two most distant but connected nodes on the graph. The graph can be deconstructed into a set of connected components where an unconnected node would form a single element of this set. The *largest connected component* is the largest element of this set. The number of *isolated* and *pendant vertices* are respectively those with no connections (*k*_*i*_ = 0) or a single connection (*k*_*i*_ = 1). The *average path length L* is the average number of nodes to connect any two (connected) points in the largest connected component of the graph:

$$L=\sum_{s,t\in V}\frac{d(s,t)}{n(n-1)}$$ (1)

where *V* is the set of nodes in the graph *G*, *d*(*s*, *t*) is the shortest path through any of the nodes in the graph from node *s* to node *t* and *n* is the number of nodes in *G*. For graphs that are not completely connected (e.g the graphs without rRNAs), we obtain the *smallest path length for largest connected subgraph*. The *clustering coefficient* of a node *s* in *G* is a measure of the interconnectivity with its neighbors. The definition in Watts and Strogatz [45] can be generalized as [51]:

CC(G)=1|V′|∑v∈V′1/3∑v∈V|{{u,w}∈E:{v,u}∈Eand{v,w}∈E}|(d(v)2) (2)

where the numerator is the average number of triangles of a node *v* and the denominator is the number triples for the node *v*, where a triple at a node *v* is defined as a path of length two where *v* is the center node. The *density* of a network is the number of edges *m* over the total possible number of possible edges ((n2) where *n* is the number of nodes):

density=m(n2) (3)

The *assortativity r* of a network is the Pearson correlation coefficient, which is a measure of the correlation between parameters, in this case degrees. We can define *e*_*ij*_ to be the fraction of edges that connect a node with *i* edges to one with *j* edges. We then define *a*_*i*_ = ∑_*j*_*e*_*ij*_ and *b*_*j*_ = ∑_*i*_*e*_*ij*_. The assortativity is [52]:

$$r=\frac{\sum_{ij}(e_{ij}-a_{i}b_{j})ij}{\sigma_{a}\sigma_{b}}$$ (4)

where *σ*_*a*,b_ is the standard deviation of the distribution of *a*, *b*. The results for all of the structural files considered are given in Table 2.

Our analysis of the small world properties is given in Table 3 for the pre-accommodated ribosome of *E*. *Coli* (pdb 5we4). A corresponding regular graph was created by defining *n* nodes equivalent to the number of nodes in the pdb 5we4 and connecting each node to its nearest unconnected neighbor using periodic boundary conditions. Note that the cluster coefficient is: 〈*k*〉/*n* where 〈*k*〉 is the average degree of the graph and *N* is the number of nodes in the network. Random graphs are Erdos-Renyi or binomial graphs with probability of attachment p=m(n2) [53]. The values in the second column of Table 3 are the average result of 5000 Erdos-Renyi random graphs. The average shortest path length was obtained from the largest connected component. Note that the average degree of the network is by definition maintained in the regular and random graphs. Similar small world characteristics were also observed for the other pdb files.

**Table 3:** Graph properties of the decoding ribosome of *E*. *Coli*, and corresponding random and lattice graphs.

	5we4	random	regular
**State**	pre-accomodation	N/A	N/A
**Species**	*E*. *Coli*	N/A	N/A
**Isolated**	0	0.048	0
**Pendant**	1	0.348	1
**Ave degree**	6.912	6.920	6.912
**Max degree**	23	13.340	8
**Diameter**	5	4.280	12
**Largest connected component**	1	1.000	1.000
**Ave path length**	2.540	2.368	5.530
**Cluster coefficient**	0.438	0.102	0.618
**Assortativity**	-0.309	-0.030	0.768
**σ = (C/C**_**rand**_**) / (L/L**_**rand**_**)**	4.019		
**ω = (L**_**rand**_**/L—C/C**_**reg**_**)**	0.224		

The ribosome network has a similar average path length as the random graph and a cluster coefficient closer to the regular graph. The small world coefficient σ and the small world measure, ω, given in Table 3 (see methods for definitions), both fall within the definition of a small world network. (σ > 1 and ω ~ 0) Note that the random and lattice graphs were generated using the same number of nodes and edges in the ribosome structural file.

**Fig 3:**
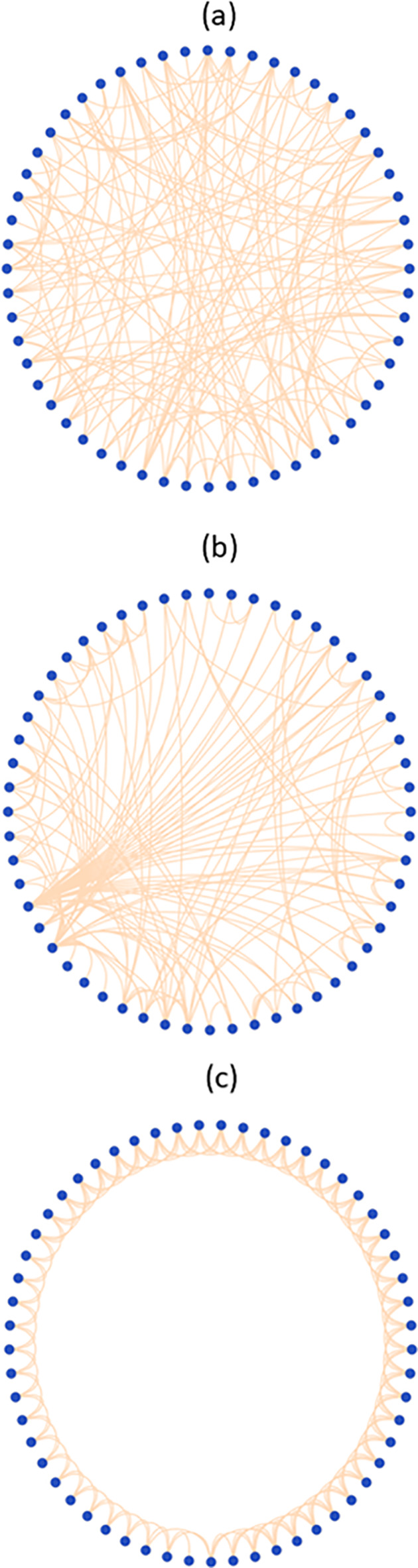
Idealization of (a) a random graph with the same connectivity and edges as in the accommodated state (4y4p), (b) the accommodated state (4y4p) and (c) a regular graph with the same connectivity as in (b) but created by connecting nodes only to nearest neighbors. Note that the rRNAs have been included as a single element in order to improve the clarity of the graphs.

To visualize the differences in these networks graphically, we prepared Fig 3 based on the accommodation step (pdb 4y4p) where each node is positioned equidistantly on a circle. To facilitate the interpretation for the reader, we have simplified the visual representation, to include just a single rRNA connection per molecule (eg we have not included the rRNA domains). The graphs are mean to provide visual representative of the differences between regular, random and small world networks. Graphically we observe the ribosomal network is clearly between a regular graph, because many connections do involve nearest neighbor interactions, and a random graph, because the presence of a few nodes with many connections provide many shortcuts.

We can also explore the small world properties by considering two quantitative parameters. The small world coefficient σ is defined by [54]:

$$\sigma =\frac{\frac{CC}{{CC}_{rand}}}{\frac{L}{L_{rand}}}$$ (5)

Networks are small world if σ > 1. Small world networks have large CC like lattices and small *L* like random graphs. The drawback of σ, however, is that it does not consider a comparison with regular graphs. One can therefore use the small world measure ω [55]:

$$\omega =\frac{L_{rand}}{L}-\frac{CC}{{CC}_{reg}}$$ (6)

If the network is ‘lattice like’, we expect *L* >> *L*_rand_, ω = -1, and if it is ‘random’ we expect *CC* >> *CC*_reg_ and ω = 1. A small-world network can roughly be assigned for -0.5 < ω < 0.5.

Centrality measures are meant to quantitatively capture the important nodes in the network. Degree centrality is a measure of communication potential, with a larger value indicating more communication. For a node *v*, it is simply the number of connections of that node normalized by the maximum possible degrees on a simple graph (which is just one less than the number of nodes *n*):

$$Degree\;centrality(v)=\frac{\#of\;connections\;to\;v}{n-1}$$ (7)

*Eigenvector centrality* measures the importance of a particular node’s connections and incorporates it into the measure. It is determined using the equation ***Ax*** = *λ****x*** where ***A*** is the adjacency matrix and λ is the eigenvalue corresponding to of the eigenvector **x** for a given node. While degree and eigenvector centrality are based on the connectivity of the nodes, another measure that can be used is based on the mean distance from one node to all other nodes. *Closeness centrality* is a measure of how close a node is to connecting to all the other nodes in the network. If *L*(*v*,*u*) is the shortest distance, measured in number of nodes, between a node *v* and *u* then we define:

$$Closeness\;centrality(v)=\frac{n-1}{\sum_{v}^{n-1}L(v,u)}$$ (8)

*Betweenness centrality* is a measure of how many linking paths a particular node makes to link two other nodes in the network. It therefore indicates the influence (control) of a node in how information is transmitted. For nodes *s*,*t*∈*V*, we define the number of paths that link them together to be *σ*_st_. For a node *v*∈*V*, we define the number of paths linking *s* and *t* that pass *v* on the way as *σ*_*st*_(*v*). Betweenness centrality is defined as:

$$Betweenness\;centrality(v)=\sum_{s,t\in V}\frac{\sigma_{st}(v)}{\sigma_{st}}$$ (9)

All centralities are calculated using the Networkx toolbox.

For a given network, the modularity *Q* [8] when divided into groups *g*_*i*_ and g_*j*_ is:

$$Q=\frac{1}{2m}\sum_{ij}B_{ij}\;\delta_{g_{i}g_{j}}$$ (10)

where $B_{ij}=A_{ij}-\frac{k_{i}k_{j}}{2m},\;A_{ij}$ is an element of the adjacency matrix, *k*_x_ is the degree of node x, and $\delta_{g_{i}g_{j}}$ is the Kronecker delta. To determine the modular groups, a Clauset-Newman-Moore greedy modularity maximization optimization is performed so that *Q* is maximized for a given set of *B*_ij_ [56]. A single modularity decomposition means that a single set of subnetworks was determined from the entire network. Subsequent decompositions involve running the optimization separately on each of the resulting subgroups.

## Results and discussion

The summary of the network properties given in Table 2 provides an overview of the four ribosomal states. We have included the counts of different types of connections: rRNA-rRNA, rprotein-rprotein, rRNA-rprotein and the connections involving mRNA and tRNA. Not surprisingly, the rRNA-rprotein connections alone form more than 50% of the network, many of which are formed during ribosome biogenesis. About 25% of the network is formed from rprotein-rprotein connections. The large number of rRNA connections as opposed to other elements results in a dis-assortative network (assortativity < 0).

Fig 2 depicts how these different interactions change with elongation stage. Note that this graph compares percentages of changes. The results from our analysis indicate that rprotein-rprotein connections change more between species compared to different states of elongation but that RNA-protein connections change more between states. This suggests that the rprotein connections may provide unique functionality or specialization. The largest source of variation between different stages is due to the rprotein-RNA connections and more specifically rRNA-rproteins.

Nevertheless, the total number of changes in the ribosome, either inter-species or at different phases, are very similar (~ 15%). Further work might explore whether there is maximum amount of changes that are acceptable for proper ribosome functioning. One of most commonly observed changes involves modifications in inter-sub-unit bridges. This may explain why this analysis revealed many unknown intersubunit connections. Such connections are critical for allowing the ribosome to continue along its path of elongation and may also provide an important mechanism for diversity in the relatively well-conserved ribosomal structure.

### The ribosomal connectome as a graph

We now consider the small world nature of the networks by considering the average path lengths, which, at ~2.5, are very small, and the cluster coefficients, which are very high. This reflects the high connectivity of the rRNAs. The comparison (Table 3) with the simulated regular graph reveals a much larger average path length (5.5) an also a slightly larger cluster coefficient. As might be expected for a small world network, the average random graph has a similar average path length as the ribosome network, but a much smaller cluster coefficient. The difference can be seen graphically in Fig 3 which compares 4y4p (accommodated state), a corresponding regular graph with nearest neighbor connections and an example of a random graph. We clearly observe that the ribosomal network is between a regular graph, because many connections do involve nearest neighbor interactions, and a random graph, because the presence of a few nodes with many connections provide many shortcuts. The quantitative parameters σ and ω, given in Table 3 (see methods for definitions), both fall within the definition of a small world network. (σ > 1 and ω ~ 0). These results clearly indicate that the bacterial ribosomal network can be classified as small world. This validates our use of network analysis to understand the properties of the ribosome connectome.

### Definition of the network

An important consideration when applying network theory to the ribosome connectome is the definition of the network. An intriguing earlier investigation represented the network on a much fine scale using either the nucleotides and/or amino acids as nodes [5] also found small world characteristics. The paper focused on the centralities of the network formed by the rRNAs and found that high centralities corresponded to nucleotides that play a critical functional role. The paper proposed that it could help to distinguish weak versus deleterious types of mutants. A more recent study defined the network using the phosphorus atom in the nucleotide and the alpha-carbon in the amino acid [6]. The centrality analysis highlighted how high betweenness centralities corresponded to an important functional role in allosteric communication pathways, implying that network analysis can reveal pathways between allosterically linked regions and possible targets for new antibiotics.

An important difference between these relatively fine-grained networks and those in this paper lies in the importance of the ribosomal proteins. By defining a network at the nucleotide or amino acid level the role of the rRNAs is emphasized because there are many more nucleotides than amino acids. In contrast, by defining the network as interconnections of rRNA domains and rproteins, the relevance of rproteins can be explored. We thus find that these two network definitions probe different characteristics of the ribosome and are complementary.

What happens if the rRNAs or the rproteins are excluded from the network? We consider the network defined by excluding the rRNAs (rproteins) and considering only edges containing at least one r-protein (rRNA) connection. Table 4 compares the network properties of the entire ribosome as well as each individual subunit using these three different methodologies for the accommodated state (pdb 5we4). Note that a similar analysis of the network with just rproteins and the sub-units has in part been carried out in previous research [7]. The first important difference is that, unlike the networks containing rRNAs, the rprotein only definition results in networks that are not completely connected. Naturally we also observe that the densities are much higher in the network that includes rRNA. For both network definitions, the ribosome is less dense than either individual subunit and the small subunit denser than the large subunit, due to the lower connectivity between the subunits. The cluster coefficient is much larger in the network with rRNAs, with the 50S subunit being larger than that of the small subunit whereas without rRNAs it is the contrary. This implies that rRNA plays a greater role in the connectivity in the large subunit, as observed already in structural investigations. Nevertheless, the rRNA only network has a cluster coefficient of two and is significantly enhanced by the presence of rproteins indicating that they play an important role in communication.

**Table 4:** Comparison of the networks found in the ribosome, large and small subunits using different definitions.

	w/rRNAs			w/o rRNAs			w/o rproteins		
	5we4	30S	50S	5we4	30S	50S	5we4	30S	50S
**Size**	68	28	43	51	22	32	23	11	15
**Order**	235	83	133	77	35	36	27	13	15
**Isolated**	0	0	0	0	0	0	0	0	0
**Pendant**	1	0	1	8	3	9	13	5	9
**Ave degree**	6.912	5.929	6.186	3.020	3.182	2.250	2.348	2.364	2.000
**Max degree**	23	16	21	7	7	4	7	7	7
**Diameter**	5	4	4	11	7	10	6	3	5
**Largest connected component**	1.000	1.000	1.000	0.961	1.000	0.813	1.000	1.000	0.867
**Ave path length**	2.536	2.029	2.209	4.664	2.835	4.640	2.957	2.055	2.641
**Cluster coefficient**	0.438	0.511	0.449	0.312	0.263	0.214	0.114	0.331	0.000
**Density**	0.103	0.220	0.147	0.060	0.152	0.073	0.107	0.236	0.143
**Assortativity**	-0.304	-0.312	-0.332	0.249	0.052	0.199	-0.682	-0.501	-0.641

In the first series we included all interactions and in the second we excluded all rRNA interactions, in the third we exclude all rprotein interactions. The small subunit is denser than the large subunit both in terms of RNAs and rproteins. The rprotein network is assortative while the rRNA and entire network is dis-assortative. The large subuit is not fully connected for the rRNA or the rprotein network.

The assortativity is also significantly different. Without rRNAs, the large sub-unit is assortative but the average degree is not even 2. Without rRNAs the assortativey of ~ 0 in the small subunit, suggesting it has random graph-like properties. The rRNA network is even more dis-assortative than the complete ribosome due to the different number of connections of the different RNAs. In the ribosome the dis-assortativity, thus, not surprisingly, arises different constituents with different properties and different functional roles. While social networks are assortative, information processing circuits such as neuronal networks and electronic circuits as well as all biological networks are in general dis-assortative.

This brief comparison shows that the communication is enhanced by the different elements in the ribosome. It suggests that including all of the elements in an initial network definition is important for network analysis and that excluding some of them (either rproteins or rRNAs) could result in misleading generalizations. The observed changes in the density and assortativity between the subunit and the entire structure, due to the intersubunit bridges, highlights the pivotal role of dynamics in the analysis. It hints that communication in this context may be dominated by mechanics, vibrational motions and allosteric pathways [30–32, 40].

### Centrality measures

**Fig 4:**
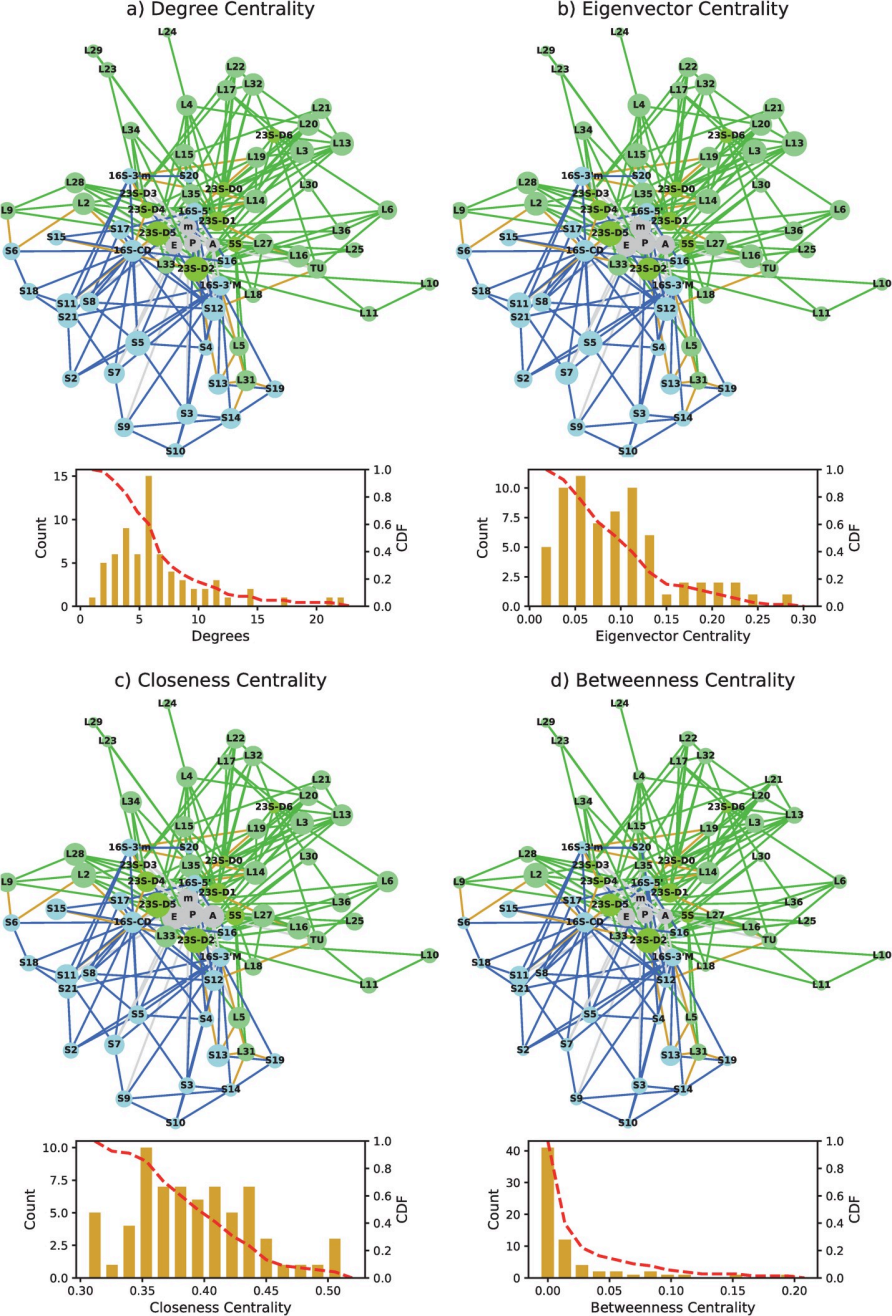
Comparison of the different centrality measures for the decoding state of *E*. *Coli* (pdb 5we4). The size of the nodes in the graphs is indicative of the centrality measure and histograms provide the distribution and the cumulative distribution function (CDF). (a) degree centrality (b) eigenvector centrality, (c) closeness centrality and (d) betweenness centrality. The small subunit nodes are depicted in blue, the large subunit nodes in green and the mRNA and tRNAs are in gray. Intersubunit bridges are in gold.

Fig 4 shows a pictorial representation of the centrality measures for the decoding state of *E*. *Coli* (pdb 5we4), where the size of the nodes is proportional to the value of centrality. The distribution of each measure is also depicted. The 15 highest score nodes, ordered by importance, for each of the elongation steps are given in Table 5. The complete list of nodes and all of their centrality scores are provided in S5–S8 Tables and the equivalent pictorial representation for the other pdb files in S1–S4 Figs. We clearly observe scale-free like behavior distribution in the degree and betweenness centrality measures, which exhibit has long tails. These are clearly associated with the measures for the rRNAs. Care should be taken when comparing hubs between the different centrality measures because the distributions are quite different.

**Table 5:** Proteins with the 15 maximum centrality scores in the networks.

**Degree Centrality**					**Eigenvector Centrality**				
**5we4**	**4v5g**	**4y4p**	**4v9h**	**4v5f**	**5we4**	**4v5g**	**4y4p**	**4v9h**	**4v5f**
23SrRNA-D2	23SrRNA-D2	23SrRNA-D2	23SrRNA-D2	23SrRNA-D2	23SrRNA-D5	23SrRNA-D5	23SrRNA-D2	23SrRNA-D2	23SrRNA-D2
23SrRNA-D5	23SrRNA-D5	23SrRNA-D5	23SrRNA-D5	23SrRNA-D5	23SrRNA-D2	23SrRNA-D2	23SrRNA-D5	23SrRNA-D5	23SrRNA-D5
16SrRNA-CD	16SrRNA-CD	16SrRNA-CD	16SrRNA-CD	16SrRNA-CD	tRNA-E	mRNA	23SrRNA-D0	23SrRNA-D1	EF-G
16SrRNA-3'M	23SrRNA-D1	23SrRNA-D0	23SrRNA-D1	EF-G	tRNA-P	tRNA-A	tRNA-P	L13	tRNA-P
23SrRNA-D1	16SrRNA-3'M	16SrRNA-3'M	EF-G	16SrRNA-3'M	mRNA	tRNA-P	tRNA-A	EF-G	23SrRNA-D1
16SrRNA-D5'	mRNA	tRNA-P	16SrRNA-3'M	23SrRNA-D1	16SrRNA-CD	tRNA-E	L15	L32	L13
mRNA	16SrRNA-D5'	23SrRNA-D1	tRNA-PE	16SrRNA-D5'	16SrRNA-3'M	S13	L20	L3	L32
tRNA-P	tRNA-E	16SrRNA-D5'	16SrRNA-3'm	tRNA-P	23SrRNA-D1	23SrRNA-D4	L32	23SrRNA-D0	L14
tRNA-E	tRNA-P	tRNA-A	16SrRNA-D5'	tRNA-E	tRNA-A	16SrRNA-CD	L27	L15	L3
23SrRNA-D0	23SrRNA-D4	tRNA-E	23SrRNA-D0	5SrRNA	L13	16SrRNA-3'M	23SrRNA-D1	5SrRNA	tRNA-E
5SrRNA	S13	23SrRNA-D3	23SrRNA-D6	23SrRNA-D0	23SrRNA-D4	L14	L13	L14	5SrRNA
16SrRNA-3'm	tRNA-A	mRNA	5SrRNA	L3	5SrRNA	L3	L3	L4	23SrRNA-D0
23SrRNA-D6	16SrRNA-3'm	5SrRNA	L14	L32	16SrRNA-3'm	16SrRNA-3'm	L4	L20	L15
23SrRNA-D4	5SrRNA	L15	23SrRNA-D4	S13	16SrRNA-D5'	23SrRNA-D1	L16	tRNA-PE	16SrRNA-CD
tRNA-A	L3	23SrRNA-D4	S5	23SrRNA-D4	L15	16SrRNA-D5'	tRNA-E	23SrRNA-D6	mRNA
**Closeness Centrality**					**Betweenness Centrality**				
23SrRNA-D5	23SrRNA-D2	tRNA-P	23SrRNA-D5	23SrRNA-D2	23SrRNA-D2	23SrRNA-D2	23SrRNA-D2	23SrRNA-D2	23SrRNA-D2
23SrRNA-D2	23SrRNA-D5	23SrRNA-D2	23SrRNA-D2	EF-G	23SrRNA-D5	23SrRNA-D5	23SrRNA-D5	23SrRNA-D5	23SrRNA-D5
tRNA-E	tRNA-A	tRNA-A	EF-G	23SrRNA-D5	16SrRNA-CD	16SrRNA-CD	16SrRNA-CD	EF-G	EF-G
tRNA-P	tRNA-P	23SrRNA-D5	tRNA-PE	tRNA-P	23SrRNA-D1	16SrRNA-3'M	16SrRNA-3'M	tRNA-PE	16SrRNA-CD
tRNA-A	S13	tRNA-E	L14	tRNA-E	tRNA-E	S13	tRNA-P	16SrRNA-CD	16SrRNA-3'M
mRNA	tRNA-E	23SrRNA-D0	L35	S13	16SrRNA-3'M	tRNA-A	tRNA-E	23SrRNA-D1	tRNA-E
L2	L14	L2	L2	L14	tRNA-P	tRNA-P	23SrRNA-D0	16SrRNA-D5'	tRNA-P
16SrRNA-CD	L3	L27	L3	L2	tRNA-A	tRNA-E	tRNA-A	16SrRNA-3'M	S13
23SrRNA-D1	mRNA	S13	L28	L3	16SrRNA-D5'	16SrRNA-D5'	23SrRNA-D1	16SrRNA-3'm	16SrRNA-D5'
L14	L2	mRNA	S7	L16	L2	23SrRNA-D1	16SrRNA-D5'	S13	23SrRNA-D1
S11	S17	L16	L33	mRNA	5SrRNA	L3	L2	L14	L2
L16	23SrRNA-D4	L14	L6	L35	L14	23SrRNA-D4	S13	S19	L3
L3	L35	L35	16SrRNA-3'm	S17	16SrRNA-3'm	L2	5SrRNA	L2	5SrRNA
16SrRNA-3'M	L16	16SrRNA-CD	16SrRNA-CD	L27	mRNA	L14	16SrRNA-3'm	S15	L14
L35	16SrRNA-CD	L3	L32	16SrRNA-3'M	S11	mRNA	S17	5SrRNA	23SrRNA-D4

The nodes are listed from the highest score to the lowest score for each file and each centrality measure. A complete list is give in S3–S6 Tables. For clarity, some of the repeated elements are highlighted by color. The observed rprotein hubs in the small subunit include: S7, S11, S13, S15, S17, S19; and in the large subunit: EF-G, L2, L3, L4, L6, L13, L14, L15, L16, L27, L28, L32, L33, L35.

### Most important hubs

A summary of the appearance of the different elements in the different centralities is given in Table 6. 23S-rRNA D2 and D5 are consistently the most important hubs for all measures. 23S-rRNA D5 has been widely discussed because of its role in the peptide bond formation and its importance in creating the tRNA-A and tRNA-P sites. 23S-rRNA D2 plays an important role in the bridges B1a and B4. 23S-D0, which forms the entrance and early portion of the tunnel [46], receives very high centrality scores in the accommodation step (4y4p). Not surprisingly, the tRNAs are also consistently important hubs.

**Table 6:** Summary of centrality results for the top 15 centrality hubs.

	Degree	Eigenvector	Closeness	Betweenness
Elements always present	23S-D1, D2, D4, D5; tRNAs*;16S-5, CD, 3m; 5S, EF-G*	23S-D1,D2,D5,EF-G,	23S-D2, D5; tRNAs*; EF-G; L2, L14, L3,	23S-D1, D2, D5; 16S-5',CD,3m; EF-G;L2
rproteins present	-	L3,L4,L13,L14,L15,L16, L20,L27, L32, S13	L2, L14, L3, L28, L27, L32, L35, S7, S11, S13, D17	L2, L3, L14, S11, S13, S15, S17, S19
rproteins present with EF		L14**		L14
Elements present in early stages	mrNA	16S,mRNA	S11	S11
Elements present in later stages		L13, L32	16S3m	16S3m***
Elements only present in T. Thermophilus		L3		

*Appears when the element is present in the file.

**Not present E Coli pre-accomodated state.

*** In hybrid state.

This table summarizes the appearance of the different elements in Table 5. We grouped them into the six groups, described in the first column. Note that no rprotein hubs are always present in the degree centrality but that their presence in the other three measures shows how important they are despite fewer connections compared to the rRNAs.

Many important hubs change in an expected manner with the different stages. For instance, mRNA is an important hub during the early steps of elongation and then falls in importance after decoding. tRNA-E is also more important in the early stages of the elongation cycle compared to the later stages, suggesting a communication pathway that could play a role in the detachment of a tRNA from the E-site of the ribosome. Interestingly, S11, which forms part of the mRNA exit tunnel is an important hub in the early stages of closeness and betweenness. 16rRNA exhibits a similar trend in the eigenvector centrality measure, which is consistent with its importance during decoding. S13 is more important in the A/T (decoding) state compared to the other steps and correspond well with its role in decoding.

At the later steps of elongation, EF-G is consistently one of the important hubs. In addition, we observe that L14, known to play an important role in the GAC, gains in centrality scores when either EF-G or EF-TU are present on the ribosome. In the hybrid state (4v9h) L35, L33 and S7 take on additional importance because of their connectivity to tRNA-PE, whereas L16 no longer appears in the top 15 because it is not connected to the tRNA sites at this step. In the accommodated state, without elongation factors, the importance of L16 and L27 confirms their important role during peptide bond formation. We also observe that 23S rRNA-D1 is of increased importance at later stages, as well as other rproteins that could potentially play an important role in communicating between the known tunnel proteins (L4, L22, L23 and L24) and other important functional sites.

### Communication pathways

Closeness centrality highlights how ‘close’ a particular element is to all other nodes and the betweenness centrality focuses on how much a particular node is found on a communication pathway between two other nodes. Not surprisingly, the tRNAs are highly ranked on the closeness centrality, especially for *T*. *Thermophilus* and for post-peptide bond states. Table 6 clearly shows that rproteins have an important role in the network even though they make less connections compared to the rRNA domains. This suggests that rproteins may provide essential shortcuts for communication between different parts in the network. The important rproteins here are typically ones that form bridges between the large and small subunits (L2, L13, S13). These rproteins also naturally also are important in the betweenness centrality.

Many of the hubs that appear in the centrality analysis thus have well known functionalities and have in many cases been explored previously. There are some elements, however, which appear that have been much less discussed, mainly L13, L15, L32 and L35. One way to understand their functionality is to explore the functionality of their connecting elements. This is the motivation for the next section.

### Modularity analysis

**Fig 5:**
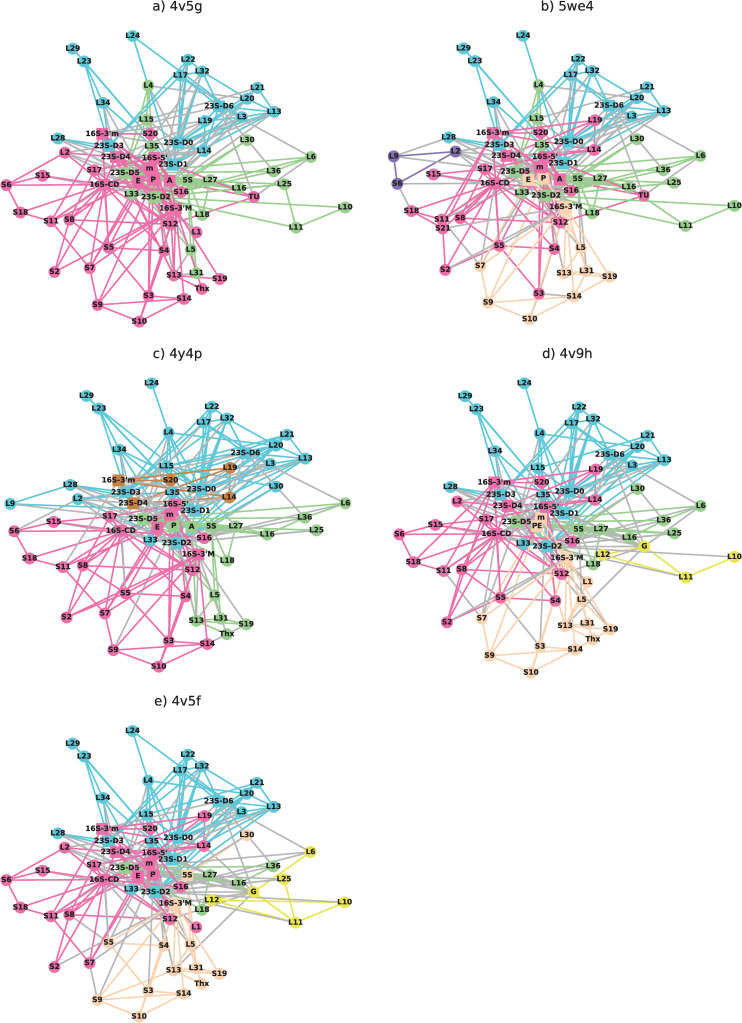
A single modularity decomposition which highlights the importance of the rRNAs. The color scheme is: green: PTC community; blue: tunnel community; pink: decoding community; peach, brown and brown: bridge communities; yellow: GAC community.

The sub-networks resulting from a first modularity decomposition are shown in Fig 5. To identify the functionality of the different sub-networks, we prepared Table 7, highlighting which elements of the ribosome are present in the different groups. A large tunnel sub-network is always present that includes 23S-D0, L22, L23 and L24. The PTC community is identified by the persistent presence of 23S-D5, L27 and L16. From these first results, we can now propose that L13 and L32 are part of the tunnel community and L15 and L35 are likely to play a role bridging the PTC and tunnel communities because they are sometimes present in one or the other.

**Table 7:** Ribosomal elements by modularity group in each of the elongation steps.

	5we4	4v5g	4y4p	4v9h	4v5f
	pre-accomodation	pre-accomodation	accomodation	hybrid	post-elongation
23SrRNA D0, D1, D3, D6 **L3**, **L13**, L17, L20, L21,L22, L23, L24, L28**, L32**, L34,	Tunnel	Tunnel	Tunnel	Tunnel	Tunnel
23SrRNA Domain 2, L4, **L15, L35**	PTC	PTC	Tunnel	Tunnel	Tunnel
23SrRNA Domain 5, **L27**, **L16**, L36, L18	PTC	PTC	PTC	PTC	PTC
5S,	PTC	PTC	PTC	PTC	bridge
L30	PTC	PTC	Tunnel	PTC	bridge
16S rRNA CD, 5' S2, S6, S8, **S11**, S12, S15, **S17**, S18	decoding	decoding	decoding	decoding	decoding
23SrRNA Domain 4, 16S rRNA 3' minor Domain S20	decoding	decoding	bridge	decoding	decoding
16S rRNA 3' Major Domain, S3, S9,S10, S14,S19	bridge	decoding	decoding	bridge	bridge
mRNA	decoding	decoding	decoding	bridge	decoding
**S7**	bridge	decoding	decoding	bridge	decoding
S4, S5	decoding	decoding	decoding	decoding	bridge
S3	decoding	decoding	decoding	bridge	bridge
**L2**	bridge 2	decoding	Tunnel	decoding	decoding
**S13, S19**	bridge	decoding	PTC	bridge	bridge
L5, L31	bridge	PTC	PTC	bridge	bridge
**L14**	decoding	Tunnel	bridge	decoding	decoding
L10, L11, (L12,G)				GAC	GAC
L6	PTC	PTC	PTC	PTC	GAC
L25	PTC	Tunnel	PTC	PTC	GAC

This table explains how we assigned the different functionalities for the first modularity decomposition. The PTC was identified by the sub-network with 23S-D5, L27 and L16; the tunnel by the presence of the maximum number of known elements that form the tunnel (23S-D0, D1, D3, D5, L3, L4, L22, L23, L24) and decoding by the presence of the maximum number of 16S rRNA domains and S12.

The pink community can be broadly labeled as ‘decoding’ because it involves 16S rRNA- D5’ and S12. In the pre-accommodated state of *E*. *Coli* as well as in the hybrid and post-peptide bond states there is an additional community, drawn in peach, that always includes S13, L5, L31 and16S-3’M, which play a role in the bridge B1. We therefore associate is as a ‘bridge’ community that connects the two subunits. Two other bridge communities also appear in two of other steps. In 5we4, a triangle between L2, L9 and S5 forms another bridge between the subunits and in 4y4p and important sub-network involves 23S-D4 and 16S-3’m and S20. Finally, in the hybrid and post-elongation states we observe a sub-network centered around EF-G.

Several interesting features emerge from this first decomposition. First, while the decoding community is dominated by elements from the small subunit, it can include elements from the large subunit such as 23S-D4, L14, L19, which are all known to participate in inter-subunit bridges. The tunnel and PTC communities only include elements from the large subunit. This difference is likely to be the result of the evolution of the ribosome, which is thought to originate with the small subunit. The tRNAs are most often found in the decoding sub-network. As a result, L1, which is thought to help the release of the tRNA from the E-site, is included in the decoding sub-network in all of the elongation steps except for the hybrid state.

The size of the communities varies with step. The PTC is much smaller after the peptide bond has been formed, the decoding community is larger at earlier stages and the bridge communities are more important at later stages of elongation. This suggests that elements which undergo a change in sub-network may either be changing functionality or providing an essential relay of information. In the latter case it would be associated with the more important sub-network, for instance the changes in sub-network of L30. Similarly, L2 connects to the mRNA exit tunnel through S6 as well as the 16S-CD and the peptide exit tunnel through 23S-D3. It may be that L2 is able to provide some information to the small subunit if the tRNA is properly accommodated.

It is interesting to compare the hybrid state, 4v9h, where the 30S subunit is rotated ~7° counterclockwise with respect to the 50S subunit [14] and the post elongation state, 4v5f. We clearly observe important differences in the connectivity related to the rotational motion and correspondingly the modularity. The head of the 30S is swiveled by 5°, which is thought to allow translocation of the tRNA from the tRNA-P site to the tRNA-E site. In the small subunit, we observe that the intersubunit connection between L2 and S6 and between S9-S13 are broken as a result. Although this does not change the first modularity decomposition we will see it does change the second one, for all four rproteins. We also observe that S13 plays a decreased role in the centrality measures in the hybrid state. In the large subunit, the L1 stalk is fully closed compared with being half closed in the canonical E-site [14]. To accommodate this, we find that there is a new connection between L1 and S13. As a result, in the hybrid state L1 belongs to the bridge community instead of decoding. Finally, we find that the conformations of EF-G in the hybid state result in a lack of connectivity between: L25-L11 and L12-L10. The EF-G community is correspondingly observed to be much smaller. It is specifically missing L6, L23, which are instead part of the PTC community. We thus see that changes due the ribosome motion can be observed in the network analysis.

### Information relays

The high centrality rproteins are likely candidates for information *relays* and indicate important nodes for information transfer. The large majority of rprotein hubs identified in Table 5 have inter-subunit connections and many also change modularity group at different steps in Table 8. Another observation is that rproteins in the small subunit tend to make relatively strong interconnections, whereas in the large subunit their connectivity is tightly linked with rRNAs. For instance, L30 makes connections to 5S and 23SrRNA in all of the files and then an additional connection to L15 in 4y4p. Except in the latter state, where it joins the L15 community to become part of the tunnel, it follows the state of 5S. Surprisingly, it is found therefore in a bridge community in 4v5f because 5S’s connection to L5 and subsequently S13. L30 could therefore potentially provide information from the tunnel via its connection to L15 in the accommodated state, to the mRNA entrance tunnel (S3, S4, S5). This analysis supports previous work considering 5S as a transducer of information [23], but shows as well that L30 could potentially works as part of its communication network.

**Table 8:** Sub-circuits observed in all 4 networks via modularity analysis with tentatively assigned functionalities.

	Decoding	Tunnel	PTC	Bridge	Misc
**Pre-accommodation**	(S5, A, m, S4, TU, 16S-5')	(L23, L24, L34, L29, 23S-D3, L28, 23S-D1)	(L15, L36, L35, L33, 23S-D5, L4, L6)	(L5, S13, S19, L31)	(L2, L9, S6)
	(S21, S20, 16S-D3'm, S2, S18, S11)				
**(5we4)**		(L20, L21, L13)	(23S-D2, L11, L10)	(16S-3'M, S7, E, P)	
***e*. *coli***	(16S-CD, S16, S15, S17, S8, S12)	(L17, L32, 23S-D0, L3, L22, 23S-D6)	(L18, L25, L30, L16, L27, 5S)	(S9, S14, S10)	
	(23S-D4, L19, L14)				
**Pre-accomodation**	(S20, A, 16S-3'm, TU, S12)	(L11, 23S-D2, L25, L10, L16)	(L15, L36, L33, L35, 23S-D5, L4, L6)		
	(S5, S4, S16, 16S-5')	(L23, L34, L29, 23S-D3, L28,23S-D1)			
	(S8, S17, S2,)	(L19, L14, L24, L3, 23S-D6)			
**(4v5g)**		(23S-D0, L20, L21, L13)	(L31, L5, L18, L30, L27, 5S)		
***T*. *Thermophilus***	(S7, L1, '16S-CD, S6, S15, S18, S11, L2, 23S-D4, E)		(23S-D2, L16, L25, L11, L10)		
		(L22, L17, L32)			
	(S9, P, 16S-3'M, S3, S14, S13, S19, S10, m, Thx)				
***Accommodation***	(S5, S2, S16, S4, S17, S8, 16S-5', S12)	(L30, L20, L15, L21, L33, 23S-D2, L35, L4, L2, 23S-D3)	(L18, L25, L16, L27, 5S)	(S20, L19, L14, 16S-D3, 23S-D4)	
	(S7, 16S-CD, S6, m, S15, S18, S11, E)				
***(4y4p)***	(S9, 16S-3'M, S3, S14, S10)	(L17, 23S-D0, L32, L3, L22, 23S-D6, L13)			
***T*. *Thermophilus***		(L23, L24, L34, L29, L9, L28, 23S-D1)	(23S-D5, L6, L36)		
	(A, P, L5, L31, S13, S19, Thx)				
**Hybrid state**	(S5, S17, S2, S4, S8, 16S-5', S12)	(23S-D0, L32, L17, L3, L22, 23S-D6, L13)	(L36, L6, 23S-D5, L25, L18, L30, L16, L27, 5S)	(L31, L5, S13, S19, Thx)	(L12, L11, G, L10)
	(16S-CD, S6, S16, S15, S18, S11)				
**(4v9h)**					
***T*. *Thermophilus***		(L20, L15, L21, L33, 23S-D2, L35, L4)		(L1, PE)	
	(S20, L19,L14, 16S-3'm, L2, 23S-D4)	(L23, L24, L34, L29, 23S-D3, L28, 23S-D1)			
**Post-peptide bond**	(S7, S18, 16S-CD, S6, S15, L2, S11)	(L20, L15, L21, L33, 23S-D2, L35, L4, L13)	(L36, 23S-D5, L18, L16, L27)	(16S-3'M, S13, Thx, S19)	(L11, L12, G, L25 L10, L6)
	(P, L1, m, 23S-D4, E)			(L5, 5S, L31, L30)	
**(4v5f)**				(S3, S5, S4)	
***T*. *Thermophilus***	(S2, S8, S17, S12)	(23S-D0, L32, L17, L24, L3, L22, 23S-D6)		(S9, S14, S10)	
		(L23, L34, L29, 23S-D3, L28, 23S-D1)			
	(S20, L19, L14, 16S-3'm, S16, 16S-5')				

The colors indicate the nodes in the first modularity decomposition. The nodes in the second modularity decomposition are placed between parenthesis and on a line when possible within the colored boxes.

L14 is also a candidate for relaying information because of its participation in different communities with functional step: decoding, bridge and the tunnel. This rprotein is an important hub in the eigenvector centrality and is part of two inter-subunit bridged (B5 and B8) connecting to the 16S rRNA [57]. Here we observe that L14 forms a path from the elongation factors to the mRNA exit tunnel. L14 plays a diminished role in *E*. *coli* data because it no longer connects to tRNA-A. L14 is the docking site for the factor RsfS [17], which blocks the joining of the subunits. Future work could explore how the connectivity of L14 changes with the docking of RsfS to ascertain its role in information transfer during its presence.

Another dominant hub is S13, which is part of an inter-subunit bridge connecting the 30S head with the 50S central protuberance, connects to L31, 23S rRNA (bridge B1a), and L5 (bridge B1b) [57, 58]. It is also likely to play the role of relay as it is observed in three different communities in Table 7, consistent with its important role in the large scale conformational rearrangements during translocation (14) in *E*. *Coli*. There is an important connectivity difference of S13 in *T*. *Thermophilus* and *E*. *coli*. S13 has two less connections in *E*. *coli* (it is not connected to tRNA-A and to Thx). A comparison of mutant *T*. *Thermophilus* with the research on *E*. *Coli* would shed light on the role of this different connectivity.

S17 is another candidate for relaying information. It also forms an intersubunit connection in three of the steps (pre-accommodation, accommodation and post-elongation) and is correspondingly a hub. It remains part of the decoding community throughout, although below in the second modularity decomposition we find that it changes sub-communities within the decoding community. It is able to play an important role because it connects to the 23S rRNA and the 16S while maintaining connections with the mRNA entrance site through its connection with S8.

Like S17, S8 and S11 remain consistently part of the decoding community. S7, the third partner of the mRNA exit tunnel, becomes part of the bridge community for the pre-accommodation state of E. Coli and in the hybrid state of T. Thermophilus. Interestingly, S11 appears in the closeness and betweenness centrality of the pre-accommodation state of E. Coli and S7 appears in the closeness centrality of the hybrid state. S7 is thus likely to play the role of relay for the mRNA exit tunnel.

### Second modularity decomposition

**Fig 6:**
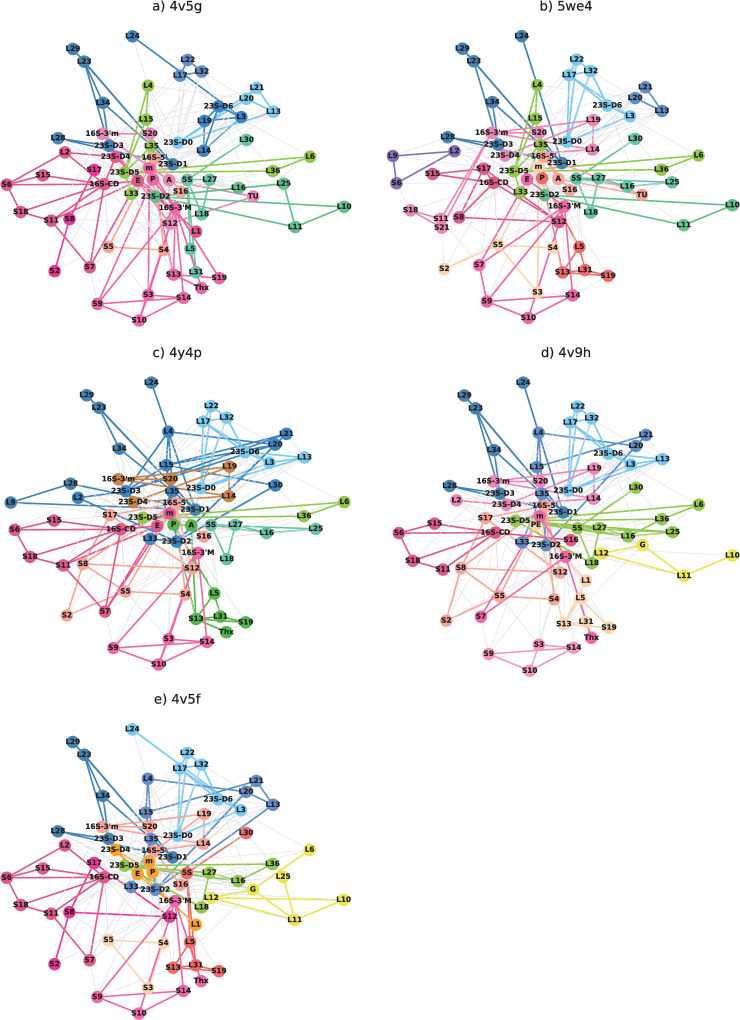
Further modularity decomposition of the networks. The decomposition is continued if the communities consist of 9 or more nodes. The color scheme is: yellow: 23S; dark blue: 5S; pink: 16S; peach: L20; turquoise: S13; brown: E; purple: L14; Orange: S2; Beige: S4-S5; navy green: EF-G or EF-TU if not in 23S community and then L10-L11 in 4v5g and L9-L28 in 4y4p; light blue-green: L22 when not included with L14; light green: L29 when not in the 23S community; light pink: S9-S10-S14 when not included with S4-S5 and S7 in 4v5f.

We consider the further modularity decomposition in Fig 6, which was carried out for sub-networks with more than 9 constituents. As a guide for the reader, in Table 8 we provide a complete listing of the elements in each decomposition. This second decomposition is more susceptible to any mistakes that are found in the structural files because a single mistake can result in a completely different second decomposition. Overall, we observe that small changes in connectivity can have large implications for sub-circuits at different stages of ribosomal function.

The second decomposition highlights the most tightly connected sub-networks that are the most likely to work together. It also indicates which sub-networks work with other sub-networks, as discussed above the 5S-L30 and the S3-S4-S5 networks. Of particular note in this decomposition is that some sub-networks of the second decomposition are found to be stable for all of the files. Two are found in the tunnel community in the large subunit: L23, L28, L29, 23S-D1, L34 and L32, L17, L22. Another is found in the small-subunit: S9-S10-S14.

Presenting both the single and the successive decompositions for the different stages suggests how small groups of ribosomal elements interact together to form functional communities. For instance, connections between the mRNA entrance and exit tunnel are connected by a tightly connected trio consisting of S9-S14-S10. Other proteins, such as L5 and L31 change functional groups at different steps. For instance, in the small subunit the second decomposition reveals communities centered typically around a single rRNA domain, suggesting that each one might serve different purposes. Some elements are often but not always found together in these smaller communities. For instance, 16S-5’ is often found with S4, S5 and S16 and 16S-CD with S6, S7, S15, S18. In comparison the PTC changes in size as function of elongation step and 23S-D5 is often just associated with L6 and L26. L33, L15 and L35 form important links between the tunnel (connections to L4), the PTC (connections to 23S-D5), are also often found in the same community,

### Relation with biogenesis

Ribosome biogenesis and the subsequent synthesis of proteins work together in an auto-regulatory feedback, resulting in a control of the number of r-proteins and the availability of rRNA [59, 60]. Assembly progresses in a hierarchal manner and assembly maps of *E*. *Coli* were first developed *in vitro* [61–63]. Subsequent research explored assembly kinetics and classified proteins according to when they are assembled [64, 65]. Others have modeled the binding energies and predict the assembly map [66]. In the small subunit, the proteins are grouped into primary binders: (16S-5D’ binding to S17, S4, S20; 16S-CD binding to S8, S15; and later 16S-3’ binding to S7), secondary binders (S12, S16, S18, S6, S9, S19, S13) and tertiary binders (S14, S10, S3, S2, S21). The assembly maps thus include the dependencies of the different proteins during assembly. A similar assembly map for the large subunit has also been developed [63], however, the assembly is more complex and has been recently explored both using cryo-EM during *in vitro* reconstitution [67] and *in vivo* using quantitative mass spectroscopy [65]. In the large subunit the earliest state has 23S rRNA domains 0, I, III, VI and the proteins L4, L13, L20, L22, L24 already assembled. The 3 protuberances and the PTC region require this core to assemble. First, L15 is a critical assembly rprotein and essential of the formation of the central protuberance and interacts functionally with L33. L5, L15 and L18 mediate the assembly of 23S and 5S. Late assembly proteins include L16, L27, L28, L33, L35 and L36 [67].

While a complete exploration of biogenesis and the networks in the ribosome would require an extensive investigation, here we provide some basic observations. First, the important hubs shown in Table 5 consist of an equal amount of primary binders (S17, S7) and secondary binders (S11, S13), however the secondary binders are in general more important hubs. In the large subunit the situation is more complex and a primary binding protein does not necessarily occur early in the binding hierarchy. Using the *in vivo* assembly, the hierarchy is divided into 6 assembly groups [65]. L14 is a primary binder for instance but does not assemble until the 4^th^ stage. Similarly, two other hubs, L2 and L19 (secondary binders) assemble in this stage as well but follow a much more complicated dependence on other proteins. The earliest hub to assemble is L5 (primary binder), in the third stage, and the others assemble: L16 (primary), L27 (secondary), L31 (secondary) assemble in the last stage. Thus in the large subunit the hubs correspond to later assembled proteins. Thus overall, we see that the important hubs in both sub-units tend to be later assembled proteins.

Concerning the relation between the communities and biogenesis, we observe that the partners in the early stages tend to belong to the same community, likely forming a core functionality and/or important basis for intercommunication between communities. In the small subunit we find 16S-CD with S15 and 16S-5’ with S4 or S20. Other interdependencies from biogenesis may or may not be present. For instance, S17 is a primary binder and is necessary for the binding of S5 and S12. In the 4 networks in Fig 6 we observe that S17 and S12 form what appears to be a permanent connection but that S17 does not connect with S5.

In the large subunit, the earliest formation includes domains 0, I, III, and VI of the 23S, which are all consistently found in the tunnel community of the first modularity decomposition. In the second decomposition a stable community 23S-D6 is often found with 23S-D0 and some of the rproteins from the central core formation: L3, L22 and L24. The assembly of 5S with L18 leaves them consistently in the same community and similarly L15 and L33 and found consistently together. As in the small subunit some of the interdependencies between rproteins formed during assembly are important in the context of the ribosome networks, however it appears to be even less the case than in the large subunit. For instance, L17 binds to 23S rRNA and interacts with L20, L22, L23, L3, L15, L27, L28, L16, L2, L32 and L19 during assembly and is an important node in the ribosome networks. However, it connects to just L22, L32 and L3. Similarly, one of the most important hubs is L14, which has few interactions during assembly.

A complete comparison of interactions that occur in biogenesis and are also found in the ribosome networks may provide information about which elements in the network play a strictly stabilization role and which are more likely to be engaged in information transfer and processing.

## Conclusions

This analysis highlights the importance of the connectivity within the ribosome and shows that it can be modeled mathematically using network theory. We find that the graphs are dis-assortative and exhibit small world properties. The changes in the rRNA connectivity and in the r-protein-r-proteins are larger between species than in different phases of translation, although overall the number of changes during elongation versus different species in the same state seem to be similar (~ 13–15% of all connections). The changes are significant and lead to different hubs and to different modular groups. The analysis here shows how small changes in connectivity can have a large impact on network properties and thus provides a possible additional mechanism for such specialization. This idea is closely related to the ribosome filter concept [68, 69] and the notion of heterogeneity [34]. This notion that the characteristics of ribosomes can result in special properties to optimize adaptation to a particular environment is an important topic in current research.

The small changes in the networks during elongation have strong implications in the dominant protein hubs and sub-networks, suggesting that this analysis might be able to provide new insights into the importance of different proteins at various stages of ribosome functioning. The modularity analysis revealed several sub-circuits where many constituents remain the same during the different stages and the two species. We focused on how some elements may serve as a relay for information from or towards important functional sites. While previous experiments probing the functionality of r-proteins typically focused on understanding one protein at a time, to test group functionality particular connections within a community could, for instance, be selectively removed.

This analysis could have broad implications. First, this analysis could be widely applicable to other types of biological nanomachines with inhomogeneous elements. Next, mathematically the networks clearly demonstrate the ability to transmit information through the various connections, but if and how it does this would still require a great deal of experimental proof. Is there a single mechanism that allows such a transfer to occur, for instance allosteric effects, as has been previously suggested [70] or could electrostatic interactions also play a role [7]? Connectivity networks have been noticed to become substantially more important with evolution [2, 7]. Could such networks provide additional regulatory functionality [71]? Analyzing communication within the ribosome and its impact on translation contributes to understanding the gene regulatory networks where the behavior of individual ribosomes are typically modeled as identical entities [72]. Such questions should be explored both with complementary mathematical analysis and biological experiments.

## Supporting information

S1 Table Numbering of the nucleotides corresponding to the different domains [46–48]. (PDF)

S2 Table Interactions in pdb files. Two indicates interaction present, one indicates the interaction elements are present but not the interaction, zero indicates that the interaction is not present and at least one of elements is missing in the file.
(PDF)

S3 Table Summary of previous explored inter-subunit bridges. (PDF)

S4 Table Observed inter-subunit interactions (not including those from S3 Table). (PDF)

S5 Table Degree centrality scores for all pdb files. Note that the hubs used in Table 5 are shaded.
(PDF)

S6 Table Eigenvector centrality scores for all pdb files. Note that the hubs used in Table 5 are shaded.
(PDF)

S7 Table Closeness centrality scores for all pdb files. Note that the hubs used in Table 5 are shaded.
(PDF)

S8 Table Betweenness centrality scores for all pdb files. Note that the hubs used in Table 5 are shaded.
(PDF)

S1 Fig Centrality measures for the pre-accomodated state of *T*. *Thermophilus* (pdb 4v5g). (PDF)

S2 Fig Centrality measures for the accomodated state of *T*. *Thermophilus* (pdb 4y4p). (PDF)

S3 Fig Centrality measures for the hybrid state of *T*. *Thermophilus* (pdb 4v9h). (PDF)

S4 Fig Centrality measures for the post-elongation state of *T*. *Thermophilus* (pdb 4v5f). (PDF)
