# Supplementary material for: Network theory of the bacterial ribosome
Source: PLoS One. 2020 Oct 5;15(10):e0239700. doi: 10.1371/journal.pone.0239700 (PMC7535068; doi:10.1371/journal.pone.0239700)
Supplement: S1 Table — (PDF) [file pone.0239700.s001.pdf]

S1 Table rRNA Domains

Domain	Nucleotides
23SrRNA Domain 0	562-585, 1251-1270, 1648-1678, 1990-2057, 2611-2625
23SrRNA Domain 1	1-561,2895-2906
23SrRNA Domain 2	587-1250
23SrRNA Domain 3	1271-1647
23SrRNA Domain 4	1679-1989
23SrRNA Domain 5	2058-2610
23SrRNA Domain 6	2626-2894
16S rRNA 5' Domain	1-566
16S rRNA Central Domain	567-915
16S rRNA 3' Major Domain	916-1396
16S rRNA 3' minor Domain	1397-1542
