# Supplementary material for: Network theory of the bacterial ribosome
Source: PLoS One. 2020 Oct 5;15(10):e0239700. doi: 10.1371/journal.pone.0239700 (PMC7535068; doi:10.1371/journal.pone.0239700)
Supplement: S2 Table — Two indicates interaction present, one indicates the interaction elements are present but not the interaction, zero indicates that the interaction is not present and at least one of elements is missing in the file. (PDF) [file pone.0239700.s002.pdf]

S2 Table All Interactions

	4v5g	5we4	4y4p	4v9h	4v5f
5SrRNA_23SrRNA-D1	2	2	2	2	2
5SrRNA_23SrRNA-D2	2	2	2	2	2
5SrRNA_23SrRNA-D5	2	2	2	2	2
5SrRNA_L5	2	2	2	2	2
5SrRNA_L16	2	2	2	2	2
5SrRNA_L18	2	2	2	2	2
5SrRNA_L25	2	2	2	2	2
5SrRNA_L27	2	2	2	2	2
5SrRNA_L30	2	2	2	2	2
5SrRNA_L31	2	2	2	1	2
16SrRNA-5'_23SrRNA-D4	2	1	2	1	2
16SrRNA-5'_EF-G	0	0	0	2	2
16SrRNA-5'_EF-TU	2	2	0	0	0
16SrRNA-5'_L14	2	2	2	2	2
16SrRNA-5'_L19	2	2	2	2	2
16SrRNA-5'_S3	2	2	2	2	2
16SrRNA-5'_S4	2	2	2	2	2
16SrRNA-5'_S5	2	2	2	2	2
16SrRNA-5'_S8	2	2	2	2	2
16SrRNA-5'_S12	2	2	2	2	2
16SrRNA-5'_S16	2	2	2	2	2
16SrRNA-5'_S17	2	2	2	2	2
16SrRNA-5'_S20	2	2	2	2	2
16SrRNA-5'_mRNA	2	2	2	1	2
16SrRNA-5'_tRNA-A	2	2	2	0	0
16SrRNA-CD_23SrRNA-D2	2	1	2	2	2
16SrRNA-CD_23SrRNA-D4	2	2	2	2	2
16SrRNA-CD_L2	2	2	2	2	2
16SrRNA-CD_S2	2	2	2	2	2
16SrRNA-CD_S4	2	2	2	2	2
16SrRNA-CD_S5	2	2	2	2	2
16SrRNA-CD_S6	2	2	2	2	2
16SrRNA-CD_S7	2	2	2	2	2
16SrRNA-CD_S8	2	2	2	2	2
16SrRNA-CD_S11	2	2	2	2	2
16SrRNA-CD_S12	2	2	2	2	2
16SrRNA-CD_S15	2	2	2	2	2
16SrRNA-CD_S16	2	2	2	2	2
16SrRNA-CD_S17	2	2	2	2	2
16SrRNA-CD_S18	2	2	2	2	2
16SrRNA-CD_S21	0	2	0	0	0
16SrRNA-CD_mRNA	2	2	2	2	2
16SrRNA-CD_tRNA-E	2	2	2	0	2
16SrRNA-CD_tRNA-P	2	2	2	0	2
16SrRNA-CD_tRNA-PE	0	0	0	2	0
16SrRNA-3'M_EF-G	0	0	0	1	2
16SrRNA-3'M_L31	1	2	2	1	1
23SrRNA-D0_L34	1	1	2	1	1

	4v5g	5we4	4y4p	4v9h	4v5f
16SrRNA-3'M_S2	2	2	2	2	2
16SrRNA-3'M_S3	2	2	2	2	2
16SrRNA-3'M_S5	2	2	2	2	2
16SrRNA-3'M_S7	2	2	2	2	2
16SrRNA-3'M_S9	2	2	2	2	2
16SrRNA-3'M_S10	2	2	2	2	2
16SrRNA-3'M_S13	2	2	2	2	2
16SrRNA-3'M_S14	2	2	2	2	2
16SrRNA-3'M_S19	2	2	2	2	2
16SrRNA-3'M_S21	0	2	0	0	0
16SrRNA-3'M_Thx	2	0	2	2	2
16SrRNA-3'M_mRNA	2	2	2	2	2
16SrRNA-3'M_tRNA-A	2	2	2	0	0
16SrRNA-3'M_tRNA-E	2	2	2	0	2
16SrRNA-3'M_tRNA-P	2	2	2	0	2
16SrRNA-3'M_tRNA-PE	0	0	0	2	0
16SrRNA-3'm_23SrRNA-D0	1	1	2	1	1
16SrRNA-3'm_23SrRNA-D4	2	2	2	2	2
16SrRNA-3'm_23SrRNA-D6	2	1	2	1	2
16SrRNA-3'm_EF-G	0	0	0	2	2
16SrRNA-3'm_L14	2	2	2	2	2
16SrRNA-3'm_L19	2	2	2	2	2
16SrRNA-3'm_S2	1	1	1	2	1
16SrRNA-3'm_S5	2	2	2	2	2
16SrRNA-3'm_S11	2	2	2	2	2
16SrRNA-3'm_S12	2	2	2	2	2
16SrRNA-3'm_S13	2	1	1	1	1
16SrRNA-3'm_S18	1	1	1	2	1
16SrRNA-3'm_S20	2	2	2	2	2
16SrRNA-3'm_S21	0	2	0	0	0
16SrRNA-3'm_mRNA	2	2	2	2	2
16SrRNA-3'm_tRNA-A	2	2	2	0	0
16SrRNA-3'm_tRNA-P	2	2	2	0	2
16SrRNA-3'm_tRNA-PE	0	0	0	2	0
23SrRNA-D0_L2	1	1	2	1	1
23SrRNA-D0_L3	2	2	2	2	2
23SrRNA-D0_L4	2	2	2	2	2
23SrRNA-D0_L13	2	2	2	2	2
23SrRNA-D0_L14	2	2	2	2	2
23SrRNA-D0_L15	2	2	2	2	2
23SrRNA-D0_L17	2	2	2	2	2
23SrRNA-D0_L20	2	2	2	2	2
23SrRNA-D0_L21	2	2	2	2	2
23SrRNA-D0_L22	2	2	2	2	2
23SrRNA-D0_L23	1	1	2	1	1
23SrRNA-D0_L27	1	1	2	1	1
23SrRNA-D0_L32	2	2	2	2	2
23SrRNA-D3_L15	1	1	2	1	1

	4v5g	5we4	4y4p	4v9h	4v5f
23SrRNA-D0_tRNA-A	1	1	2	0	0
23SrRNA-D0_tRNA-P	1	1	2	0	1
23SrRNA-D1_L3	2	1	1	1	1
23SrRNA-D1_L4	2	2	2	2	2
23SrRNA-D1_L9	0	1	2	0	0
23SrRNA-D1_L13	2	2	2	2	2
23SrRNA-D1_L15	2	2	2	2	2
23SrRNA-D1_L20	2	2	2	2	2
23SrRNA-D1_L21	1	2	1	2	2
23SrRNA-D1_L22	2	2	2	2	2
23SrRNA-D1_L23	2	2	2	2	2
23SrRNA-D1_L24	2	2	2	2	2
23SrRNA-D1_L28	2	2	2	2	2
23SrRNA-D1_L29	2	2	2	2	2
23SrRNA-D1_L32	2	2	2	2	2
23SrRNA-D1_L34	2	2	2	2	2
23SrRNA-D1_L35	2	2	2	2	2
23SrRNA-D1_tRNA-E	1	2	1	0	1
23SrRNA-D2_EF-G	0	0	0	2	2
23SrRNA-D2_L2	2	2	2	2	2
23SrRNA-D2_L3	2	2	2	2	2
23SrRNA-D2_L4	2	2	2	2	2
23SrRNA-D2_L6	2	2	2	2	2
23SrRNA-D2_L10	2	2	0	2	2
23SrRNA-D2_L11	2	2	0	2	2
23SrRNA-D2_L13	2	2	2	2	2
23SrRNA-D2_L15	2	2	2	2	2
23SrRNA-D2_L16	2	2	2	2	2
23SrRNA-D2_L20	2	2	2	2	2
23SrRNA-D2_L21	2	2	2	0	2
23SrRNA-D2_L22	2	2	2	2	2
23SrRNA-D2_L25	2	2	2	2	2
23SrRNA-D2_L27	2	2	2	2	2
23SrRNA-D2_L30	2	2	2	2	2
23SrRNA-D2_L32	2	1	2	2	2
23SrRNA-D2_L33	2	2	2	2	2
23SrRNA-D2_L34	2	2	2	2	2
23SrRNA-D2_L35	2	2	2	2	2
23SrRNA-D2_L36	2	2	2	2	2
23SrRNA-D2_S13	2	2	2	1	2
23SrRNA-D2_S15	2	2	2	2	2
23SrRNA-D2_S17	2	1	2	1	2
23SrRNA-D2_S19	1	1	1	2	1
23SrRNA-D2_tRNA-A	2	2	2	0	0
23SrRNA-D3_L2	2	2	2	2	2
23SrRNA-D3_L4	1	1	2	1	1
23SrRNA-D5_tRNA-E	2	2	2	0	2
23SrRNA-D5_tRNA-P	2	2	2	0	2
23SrRNA-D5_tRNA-PE	0	0	0	2	0
23SrRNA-D6_EF-G	0	0	0	2	2

	4v5g	5we4	4y4p	4v9h	4v5f
23SrRNA-D3_L17	2	2	2	2	2
23SrRNA-D3_L20	1	1	2	1	1
23SrRNA-D3_L21	1	1	2	1	1
23SrRNA-D3_L22	2	2	2	2	2
23SrRNA-D3_L23	2	2	2	2	2
23SrRNA-D3_L28	2	2	2	2	2
23SrRNA-D3_L32	1	1	2	1	2
23SrRNA-D3_L34	2	2	2	2	2
23SrRNA-D4_EF-G	0	0	0	2	2
23SrRNA-D4_L1	2	0	0	2	2
23SrRNA-D4_L2	2	2	2	2	2
23SrRNA-D4_L3	1	1	2	1	1
23SrRNA-D4_L14	2	2	2	2	2
23SrRNA-D4_L17	1	1	2	1	1
23SrRNA-D4_L19	2	2	2	2	2
23SrRNA-D4_L28	2	2	2	2	2
23SrRNA-D4_L34	2	2	2	2	2
23SrRNA-D4_S13	2	1	1	1	1
23SrRNA-D4_mRNA	2	2	1	1	1
23SrRNA-D4_tRNA-A	2	2	2	0	0
23SrRNA-D4_tRNA-E	2	2	2	0	2
23SrRNA-D4_tRNA-P	2	2	2	0	2
23SrRNA-D4_tRNA-PE	0	0	0	2	0
23SrRNA-D5_EF-G	0	0	0	2	2
23SrRNA-D5_L1	2	0	0	2	2
23SrRNA-D5_L2	2	2	2	2	2
23SrRNA-D5_L3	2	2	2	2	2
23SrRNA-D5_L4	2	2	2	2	2
23SrRNA-D5_L5	2	2	2	2	2
23SrRNA-D5_L6	2	2	2	2	2
23SrRNA-D5_L9	0	2	2	0	0
23SrRNA-D5_L13	2	2	2	2	2
23SrRNA-D5_L14	2	2	2	2	2
23SrRNA-D5_L15	2	2	2	2	2
23SrRNA-D5_L16	2	2	2	2	2
23SrRNA-D5_L18	2	2	2	2	2
23SrRNA-D5_L25	1	1	1	2	1
23SrRNA-D5_L27	2	2	2	2	2
23SrRNA-D5_L28	2	2	2	2	2
23SrRNA-D5_L32	2	2	2	2	2
23SrRNA-D5_L33	2	2	2	2	2
23SrRNA-D5_L35	2	2	2	2	2
23SrRNA-D5_L36	2	2	2	2	2
23SrRNA-D5_S7	1	1	1	2	1
23SrRNA-D5_S11	2	2	1	2	2
23SrRNA-D5_tRNA-A	2	2	2	0	0
L11_L25	2	1	0	1	2
L13_L20	2	2	2	2	2
L13_L21	2	2	2	2	2
L14_L19	2	2	2	2	2

	4v5g	5we4	4y4p	4v9h	4v5f
23SrRNA-D6_EF-TU	2	2	0	0	0
23SrRNA-D6_L3	2	2	2	2	2
23SrRNA-D6_L6	2	2	2	2	2
23SrRNA-D6_L13	2	2	2	2	2
23SrRNA-D6_L14	2	2	2	2	2
23SrRNA-D6_L17	2	2	2	2	2
23SrRNA-D6_L19	2	2	2	2	2
23SrRNA-D6_L32	2	2	2	2	2
23SrRNA-D6_L36	2	2	2	2	2
EF-G_L6	0	0	0	2	2
EF-G_L11	0	0	0	2	2
EF-G_L12	0	0	0	2	2
EF-G_L14	0	0	0	2	2
EF-G_S12	0	0	0	2	2
EF-G_S13	0	0	0	2	2
EF-G_mRNA	0	0	0	1	2
EF-G_tRNA-P	0	0	0	0	2
EF-TU_L6	2	2	0	0	0
EF-TU_S12	2	2	0	0	0
EF-TU_tRNA-A	2	2	0	0	0
L1_S13	1	0	0	2	1
L1_tRNA-E	2	0	0	0	2
L1_tRNA-PE	0	0	0	2	0
L2_L9	0	2	1	0	0
L2_S6	2	2	2	1	2
L3_L13	2	2	2	2	2
L3_L14	2	2	2	2	2
L3_L17	2	2	2	2	2
L3_L19	2	2	2	2	2
L3_L24	2	1	1	1	2
L4_L15	2	2	2	2	2
L4_L20	2	2	2	2	2
L5_L31	2	2	2	2	2
L5_S13	2	2	2	2	2
L5_S19	1	1	1	2	1
L5_tRNA-P	2	2	2	0	2
L6_L36	2	2	2	2	1
L9_L28	0	2	2	0	0
L9_S6	0	2	1	0	0
L10_L11	2	2	0	2	2
L10_L12	0	0	0	1	2
L11_L12	0	0	0	2	2
L11_L16	1	2	0	1	1
S7_S9	2	2	2	2	2
S7_S11	2	2	2	2	2
S7_mRNA	2	2	2	2	2
S7_tRNA-E	2	2	2	0	2
S8_S12	2	2	2	2	2
S8_S17	2	2	2	2	2
S9_S10	2	2	2	2	2

	4v5g	5we4	4y4p	4v9h	4v5f
L14_tRNA-A	2	1	1	0	0
L15_L20	1	1	2	1	1
L15_L21	2	2	2	2	2
L15_L30	1	1	2	1	1
L15_L35	2	2	2	2	2
L16_L25	2	2	2	2	2
L16_L27	2	2	2	2	2
L16_L36	1	1	2	2	2
L16_tRNA-A	1	1	2	0	0
L16_tRNA-P	2	2	2	0	2
L17_L22	2	2	2	2	2
L17_L32	2	2	2	2	2
L18_L27	2	2	2	2	2
L20_L21	2	2	2	2	2
L20_L32	2	2	2	2	2
L22_L32	2	2	2	2	2
L23_L29	2	2	2	2	2
L23_L34	2	1	2	2	2
L27_tRNA-A	1	1	2	0	0
L27_tRNA-P	2	2	2	0	2
L28_tRNA-E	2	2	2	0	2
L28_tRNA-PE	0	0	0	2	0
L31_S13	2	2	2	2	2
L31_S14	1	2	1	1	1
L31_S19	1	2	2	1	2
L33_L35	2	2	2	2	2
L33_tRNA-E	2	2	2	0	2
L33_tRNA-PE	0	0	0	2	0
L35_tRNA-E	2	2	2	0	2
L35_tRNA-PE	0	0	0	2	0
S2_S5	1	2	2	2	1
S2_S8	2	1	2	2	2
S2_S21	0	2	0	0	0
S3_S4	2	1	2	1	2
S3_S5	2	2	2	2	2
S3_S10	2	2	2	2	2
S3_S14	2	2	2	2	2
S3_mRNA	2	2	2	1	1
S4_S5	2	2	2	2	2
S5_S8	2	2	2	2	2
S5_mRNA	2	2	2	1	1
S6_S15	2	1	2	2	2
S6_S18	2	2	2	2	2

	4v5g	5we4	4y4p	4v9h	4v5f
**S9_S13**	1	1	1	1	2
**S9_S14**	2	2	2	2	2
**S9_tRNA-P**	2	2	2	0	2
**S9_tRNA-PE**	0	0	0	2	0
**S10_S14**	2	2	2	2	2
**S11_S18**	2	2	2	2	2
**S11_S21**	0	2	0	0	0
**S11_tRNA-E**	2	2	2	0	2
**S12_S17**	2	2	2	2	2
**S12_mRNA**	2	2	2	1	2
**S12_tRNA-A**	2	2	1	0	0
**S13_S19**	2	2	2	2	2
**S13_Thx**	2	0	2	2	2
**S13_mRNA**	2	1	1	1	1
**S13_tRNA-A**	2	1	2	0	0
**S13_tRNA-P**	2	2	2	0	2
**S13_tRNA-PE**	0	0	0	2	0
**S14_S19**	1	2	2	2	1
**S15_S17**	1	2	2	2	1
**S18_S21**	0	2	0	0	0
**mRNA_tRNA-A**	2	2	2	0	0
**mRNA_tRNA-E**	2	2	2	0	2
**mRNA_tRNA-P**	2	2	2	0	2
**mRNA_tRNA-PE**	0	0	0	2	0
**tRNA-A_tRNA-P**	1	1	2	0	0
**tRNA-E_tRNA-P**	2	2	2	0	1

	4v5g	5we4	4y4p	4v9h	4v5f
