# Supplementary material for: Network theory of the bacterial ribosome
Source: PLoS One. 2020 Oct 5;15(10):e0239700. doi: 10.1371/journal.pone.0239700 (PMC7535068; doi:10.1371/journal.pone.0239700)
Supplement: S3 Table — (PDF) [file pone.0239700.s003.pdf]

S3 Table Previously Explored Inter-subunit Bridges

	T. Thermophilus		*E.Coli*		
	30S	50S	30S	50S	pdb files observed
B1a	S13	23S-D2	S13	23S-D2	5we4, 4v5g, 4v9h, 4v5f
B1b	S13	L5, L31	S13	L5, L31	all
B1c	/	L31	S14, S19, 16S	L31	5we4, 4y4p, 4v5f
B2a/d	16S 3m, 16S CD	23S-D4	16S3m	23S-D4	all
B2b/c	16S-CD	23S-D4	16S-CD	23S-D4	all
B3	16S3m	23S-D4	16S3m	23S-D4	all
B4	S15	23S-D2	S15	23S-D2	all
B5	16S3m	23S-D4	16S3m	23S-D4	all
B6	16S3m	L19	16S3m	L19	all
B7	16S-CD	L2, 23S-4	16S-CD	L2, 23S-4	all
B8	16S-5	L14	16S-5	L14	all
RRF bound	S7	23S-D5	S7	23S-D5	4v9h
