# Supplementary material for: Network theory of the bacterial ribosome
Source: PLoS One. 2020 Oct 5;15(10):e0239700. doi: 10.1371/journal.pone.0239700 (PMC7535068; doi:10.1371/journal.pone.0239700)
Supplement: S4 Table — (PDF) [file pone.0239700.s004.pdf]

S4 Table Observed Inter-subunit Interactions*

30S	50S	pdb file observed
16S-3m	L14	all
16S-5	L19	all
S6	L2	5we4, 4v5g, 4v9h, 4v5f
S6	L9	5we4
m	23S-D4	5we4, 4v5g
S11	23S-D5	5we4,4v5g, 4v9h, 4v5f
S17	23S-D2	4v5g,4y4p, 4v5f
S13	23S-D4	4v5g
16S-CD	23S-D2	4v5g, 4y4p, 4v9h, 4v5f
16S-5	23S-D4	4v5g, 4y4p, 4v5f
16S3m	23S-D6	4v5g, 4y4p, 4v9h
16S3m	23S-D0	4y4p
S13	L1	4v9h
S19	L5	4v9h
S19	23S-D2	4v9h

* Note that the bridges from S5 are not included here
