# Supplementary material for: Network theory of the bacterial ribosome
Source: PLoS One. 2020 Oct 5;15(10):e0239700. doi: 10.1371/journal.pone.0239700 (PMC7535068; doi:10.1371/journal.pone.0239700)
Supplement: S5 Table — Note that the hubs used in Table 5 are shaded. (PDF) [file pone.0239700.s005.pdf]

S5 Table Degree Centrality

decoding *(Thermus)*		decoding (*E. Coli)*		pre-peptide bond		mid-elongation		post-elongation	
**4v5g**	**value**	**5we4**	**value**	**4y4p**	**value**	**4v9h**	**value**	**4v9f**	**value**
23SrRNA-D2	0.3731	23SrRNA-D2	0.3433	23SrRNA-D2	0.3594	23SrRNA-D2	0.3636	23SrRNA-D2	0.3731
23SrRNA-D5	0.3284	23SrRNA-D5	0.3284	23SrRNA-D5	0.3281	23SrRNA-D5	0.3485	23SrRNA-D5	0.3284
16S-rRNA-CD	0.2388	16S-rRNA-CD	0.2537	16S-rRNA-CD	0.2500	16S-rRNA-CD	0.2273	16S-rRNA-CD	0.2388
23SrRNA-D1	0.2090	16S-rRNA-3'M	0.2239	23SrRNA-D0	0.2500	23SrRNA-D1	0.2121	EF-G	0.2239
16S-rRNA-3'M	0.2090	23SrRNA-D1	0.2239	16S-rRNA-3'M	0.2344	EF-G	0.1818	16S-rRNA-3'M	0.209
mRNA	0.1940	16S-rRNA-D5'	0.1940	tRNA-P	0.2188	16S-rRNA-3'M	0.1818	23SrRNA-D1	0.209
16S-rRNA-D5'	0.1940	mRNA	0.1791	23SrRNA-D1	0.2188	tRNA-PE	0.1818	16S-rRNA-D5'	0.1791
tRNA-E	0.1791	tRNA-P	0.1791	16S-rRNA-D5'	0.1875	16S-rRNA-3'm	0.1667	tRNA-P	0.1791
tRNA-P	0.1791	tRNA-E	0.1791	tRNA-A	0.1875	16S-rRNA-D5'	0.1667	tRNA-E	0.1642
23SrRNA-D4	0.1642	23SrRNA-D0	0.1493	tRNA-E	0.1719	23SrRNA-D0	0.1515	5SrRNA	0.1493
S13	0.1642	5SrRNA	0.1493	23SrRNA-D3	0.1719	23SrRNA-D6	0.1364	23SrRNA-D0	0.1493
tRNA-A	0.1642	16S-rRNA-3'm	0.1493	mRNA	0.1719	5SrRNA	0.1364	L3	0.1343
16S-rRNA-3'm	0.1493	23SrRNA-D6	0.1343	5SrRNA	0.1563	L14	0.1364	L32	0.1343
5SrRNA	0.1493	23SrRNA-D4	0.1343	L15	0.1563	23SrRNA-D4	0.1212	S13	0.1343
L3	0.1493	tRNA-A	0.1343	23SrRNA-D4	0.1563	S5	0.1212	23SrRNA-D4	0.1343
23SrRNA-D0	0.1493	S5	0.1343	S5	0.1406	S13	0.1212	L14	0.1343
L14	0.1343	L14	0.1194	L32	0.1406	L32	0.1212	23SrRNA-D6	0.1343
23SrRNA-D6	0.1343	L13	0.1194	16S-rRNA-3'm	0.1406	L3	0.1212	16S-rRNA-3'm	0.1343
S5	0.1194	S12	0.1194	L3	0.1406	L13	0.1212	mRNA	0.1343
L13	0.1194	L3	0.1194	L20	0.1406	L20	0.1061	L13	0.1194
L32	0.1194	L32	0.1045	L13	0.1250	L15	0.1061	23SrRNA-D3	0.1045
S12	0.1194	S11	0.1045	L16	0.1250	L4	0.0909	S12	0.1045
L15	0.1045	L2	0.1045	L14	0.1250	L21	0.0909	L15	0.1045
S3	0.1045	L20	0.1045	L27	0.1250	L16	0.0909	S5	0.1045
L20	0.1045	L15	0.1045	23SrRNA-D6	0.1250	L35	0.0909	L16	0.1045
L4	0.0896	L16	0.1045	S13	0.1250	23SrRNA-D3	0.0909	L20	0.1045
L19	0.0896	23SrRNA-D3	0.0896	L2	0.1094	L19	0.0909	L19	0.0896
L27	0.0896	S13	0.0896	L4	0.1094	S7	0.0909	L21	0.0896
L16	0.0896	S3	0.0896	S3	0.1094	L17	0.0909	S7	0.0896
L2	0.0896	S21	0.0896	L17	0.1094	L22	0.0909	L22	0.0896
S7	0.0896	L31	0.0896	S8	0.0938	S8	0.0909	L4	0.0896
23SrRNA-D3	0.0896	L35	0.0896	S17	0.0938	S12	0.0909	L2	0.0896
L35	0.0896	S7	0.0896	S12	0.0938	L27	0.0758	S3	0.0896
S8	0.0896	S14	0.0896	S7	0.0938	L2	0.0758	S9	0.0896
L22	0.0896	L21	0.0896	L21	0.0938	S17	0.0758	S8	0.0896
L17	0.0896	L4	0.0896	L19	0.0938	S19	0.0758	S11	0.0896
S11	0.0896	L19	0.0896	L28	0.0938	L36	0.0758	L35	0.0896
L34	0.0746	L17	0.0896	L35	0.0938	L34	0.0758	L17	0.0896
L6	0.0746	L22	0.0896	L22	0.0938	S2	0.0758	L27	0.0896
L28	0.0746	L27	0.0896	L34	0.0938	S14	0.0758	L34	0.0746
L5	0.0746	L28	0.0896	L31	0.0781	L5	0.0758	S17	0.0746
EF-TU	0.0746	S8	0.0746	S9	0.0781	S11	0.0758	L28	0.0746
S17	0.0746	S9	0.0746	L36	0.0781	S9	0.0758	L11	0.0746
S9	0.0746	L5	0.0746	L23	0.0781	L6	0.0758	L5	0.0746
L21	0.0746	L6	0.0746	L5	0.0781	S3	0.0758	L33	0.0597

decoding *(Thermus)*		decoding (*E. Coli)*		pre-peptide bond		mid-elongation		post-elongation	
**4v5g**	**value**	**5we4**	**value**	**4y4p**	**value**	**4v9h**	**value**	**4v9f**	**value**
L33	0.0597	S17	0.0746	S11	0.0781	mRNA	0.0758	S14	0.0597
L23	0.0597	EF-TU	0.0746	S14	0.0781	L28	0.0758	L23	0.0597
L25	0.0597	L34	0.0597	S6	0.0625	L33	0.0606	L36	0.0597
L36	0.0597	S6	0.0597	S15	0.0625	S18	0.0606	S10	0.0597
S6	0.0597	S18	0.0597	S4	0.0625	S10	0.0606	S6	0.0597
S14	0.0597	L36	0.0597	S10	0.0625	L25	0.0606	L6	0.0597
S10	0.0597	S2	0.0597	S19	0.0625	L1	0.0606	S4	0.0597
S4	0.0597	L9	0.0597	L33	0.0625	L23	0.0606	L31	0.0597
S2	0.0448	S19	0.0597	S2	0.0625	L11	0.0606	L25	0.0597
L11	0.0448	L33	0.0597	L6	0.0625	S15	0.0606	S2	0.0448
L1	0.0448	S10	0.0597	L18	0.0469	S6	0.0455	L10	0.0448
L18	0.0448	L11	0.0448	S18	0.0469	L18	0.0455	L1	0.0448
L31	0.0448	S15	0.0448	L9	0.0469	S4	0.0455	L18	0.0448
S18	0.0448	S4	0.0448	L25	0.0469	L31	0.0303	L12	0.0448
S15	0.0448	L23	0.0448	L30	0.0469	L10	0.0303	S19	0.0448
L10	0.0299	L18	0.0448	Thx	0.0313	Thx	0.0303	S18	0.0448
L24	0.0299	L25	0.0448	S16	0.0313	L12	0.0303	S15	0.0448
L29	0.0299	L29	0.0299	S20	0.0313	S16	0.0303	L24	0.0299
Thx	0.0299	S16	0.0299	L29	0.0313	S20	0.0303	L30	0.0299
S19	0.0299	S20	0.0299	L24	0.0156	L29	0.0303	Thx	0.0299
S16	0.0299	L10	0.0299			L30	0.0303	S16	0.0299
S20	0.0299	L30	0.0299			L24	0.0152	S20	0.0299
L30	0.0299	L24	0.0149					L29	0.0299
