# Supplementary material for: Network theory of the bacterial ribosome
Source: PLoS One. 2020 Oct 5;15(10):e0239700. doi: 10.1371/journal.pone.0239700 (PMC7535068; doi:10.1371/journal.pone.0239700)
Supplement: S6 Table — Note that the hubs used in Table 5 are shaded. (PDF) [file pone.0239700.s006.pdf]

S6 Table Eigenvector Centrality

decoding *(Thermus)*		decoding (*E. Coli)*		pre-peptide bond		mid-elongation		post-elongation	
**4v5g**	**value**	**5we4**	**value**	**4y4p**	**value**	**4v9h**	**value**	**4v9f**	**value**
23SrRNA-D5	0.3025	23SrRNA-D5	0.3147	23SrRNA-D2	0.3054	23SrRNA-D2	0.3535	23SrRNA-D2	0.3259
23SrRNA-D2	0.2885	23SrRNA-D2	0.2694	23SrRNA-D5	0.2958	23SrRNA-D5	0.3526	23SrRNA-D5	0.3228
mRNA	0.2229	tRNA-E	0.2263	23SrRNA-D0	0.2509	23SrRNA-D1	0.1986	EF-G	0.2500
tRNA-A	0.2166	tRNA-P	0.2159	tRNA-P	0.2199	L13	0.1963	tRNA-P	0.2001
tRNA-P	0.2137	mRNA	0.2090	tRNA-A	0.2189	EF-G	0.1957	23SrRNA-D1	0.1670
tRNA-E	0.2004	16S-rRNA-CD	0.1989	L15	0.1874	L32	0.1848	L13	0.1661
S13	0.1791	16S-rRNA-3'M	0.1866	L20	0.1735	L3	0.1818	L32	0.1646
23SrRNA-D4	0.1775	23SrRNA-D1	0.1794	L32	0.1732	23SrRNA-D0	0.1811	L14	0.1636
16S-rRNA-CD	0.1749	tRNA-A	0.1780	L27	0.1707	L15	0.1753	L3	0.1621
16S-rRNA-3'M	0.1716	L13	0.1520	23SrRNA-D1	0.1680	5SrRNA	0.1698	tRNA-E	0.1601
L14	0.1622	23SrRNA-D4	0.1505	L13	0.1633	L14	0.1672	5SrRNA	0.1553
L3	0.1613	5SrRNA	0.1504	L3	0.1629	L4	0.1614	23SrRNA-D0	0.1511
16SrRNA-D3	0.1586	16SrRNA-D3	0.1461	L4	0.1579	L20	0.1611	L15	0.1477
23SrRNA-D1	0.1527	16S-rRNA-D5'	0.1412	L16	0.1562	tRNA-PE	0.1578	16S-rRNA-CD	0.1477
16S-rRNA-D5'	0.1498	L15	0.1408	tRNA-E	0.1532	23SrRNA-D6	0.1571	mRNA	0.1456
L13	0.1451	L3	0.1407	23SrRNA-D3	0.1518	L35	0.1536	16S-rRNA-3'M	0.1448
5SrRNA	0.1394	L35	0.1368	5SrRNA	0.1468	L21	0.1437	L16	0.1433
L32	0.1359	L14	0.1353	23SrRNA-D4	0.1463	L16	0.1412	23SrRNA-D6	0.1418
23SrRNA-D0	0.1312	23SrRNA-D0	0.1329	mRNA	0.1355	L6	0.1349	L4	0.1361
L15	0.1301	L16	0.1314	L2	0.1351	L36	0.1293	L20	0.1340
L35	0.1262	L4	0.1281	16S-rRNA-3'M	0.1340	L27	0.1238	L35	0.1337
S12	0.1249	S5	0.1281	L14	0.1272	L22	0.1230	23SrRNA-D4	0.1331
L16	0.1215	L27	0.1270	16S-rRNA-CD	0.1270	L33	0.1155	L27	0.1323
L27	0.1212	S11	0.1257	L21	0.1269	L25	0.1154	S13	0.1299
L4	0.1204	L2	0.1221	L35	0.1236	23SrRNA-D4	0.1128	16SrRNA-D3	0.1255
S5	0.1179	L20	0.1200	L22	0.1198	L2	0.1121	16S-rRNA-D5'	0.1224
L20	0.1142	S12	0.1198	L17	0.1140	16SrRNA-D3	0.1116	L21	0.1190
L2	0.1114	S7	0.1123	L34	0.1128	16S-rRNA-CD	0.1041	L2	0.1135
23SrRNA-D6	0.1108	L32	0.1122	S13	0.1107	L17	0.1013	L6	0.1134
S11	0.1045	L21	0.1101	23SrRNA-D6	0.1026	L28	0.1006	L22	0.1057
S7	0.1006	L28	0.1098	16SrRNA-D3	0.1016	L19	0.0929	L33	0.1027
L19	0.0990	23SrRNA-D6	0.1055	L28	0.1002	16S-rRNA-D5'	0.0879	S12	0.1020
L33	0.0987	L33	0.1049	16S-rRNA-D5'	0.0985	L34	0.0876	L36	0.1018
L28	0.0952	S13	0.0975	L36	0.0971	S7	0.0812	S11	0.0948
L5	0.0946	L5	0.0939	L33	0.0904	L1	0.0797	L28	0.0934
L6	0.0928	L6	0.0938	L5	0.0850	S13	0.0790	L5	0.0930
L22	0.0896	L19	0.0907	L6	0.0825	16S-rRNA-3'M	0.0786	L19	0.0925
S17	0.0875	L22	0.0907	S5	0.0803	L5	0.0782	L17	0.0872
L21	0.0871	L36	0.0867	L19	0.0761	S11	0.0778	S7	0.0829
S3	0.0856	S21	0.0857	L23	0.0729	L18	0.0733	S17	0.0828
L36	0.0855	S3	0.0845	S17	0.0716	L11	0.0712	L11	0.0810
L34	0.0756	L34	0.0724	S7	0.0667	S19	0.0700	L34	0.0804
S8	0.0754	S8	0.0723	L30	0.0659	S12	0.0666	L25	0.0769
EF-TU	0.0748	EF-TU	0.0707	L18	0.0632	23SrRNA-D3	0.0644	S5	0.0757
L1	0.0732	L17	0.0705	L25	0.0627	S5	0.0606	23SrRNA-D3	0.0744

decoding *(Thermus)*		decoding (*E. Coli)*		pre-peptide bond		mid-elongation		post-elongation	
**4v5g**	**value**	**5we4**	**value**	**4y4p**	**value**	**4v9h**	**value**	**4v9f**	**value**
L17	0.0732	L31	0.0696	S12	0.0601	mRNA	0.0605	S9	0.0678
L25	0.0636	S9	0.0679	L9	0.0581	L30	0.0594	L1	0.0671
L18	0.0606	S2	0.0664	S3	0.0561	S15	0.0592	L18	0.0665
S9	0.0605	L9	0.0659	S15	0.0555	L10	0.0482	S8	0.0621
S4	0.0569	L18	0.0656	L31	0.0526	S8	0.0461	S15	0.0557
S15	0.0542	S17	0.0655	S9	0.0494	S2	0.0455	L30	0.0525
23SrRNA-D3	0.0514	L25	0.0610	S8	0.0492	L23	0.0429	S3	0.0490
L30	0.0461	S15	0.0591	S11	0.0484	S9	0.0415	L10	0.0489
S2	0.0454	S14	0.0551	S2	0.0402	S17	0.0413	L31	0.0450
L31	0.0445	23SrRNA-D3	0.0547	S4	0.0373	S18	0.0359	S4	0.0430
L11	0.0417	S4	0.0518	S6	0.0349	S3	0.0312	L12	0.0414
S6	0.0404	S18	0.0508	S19	0.0338	L12	0.0303	S2	0.0386
S14	0.0383	S6	0.0485	S14	0.0310	S4	0.0287	S6	0.0379
S10	0.0383	L11	0.0483	S10	0.0279	S14	0.0274	L23	0.0375
Thx	0.0377	L30	0.0465	Thx	0.0252	L29	0.0274	L24	0.0359
S19	0.0377	S19	0.0453	L29	0.0248	S20	0.0226	S19	0.0348
L10	0.0355	S10	0.0436	S16	0.0232	S6	0.0226	S14	0.0320
S16	0.0349	S16	0.0377	S18	0.0217	L24	0.0225	S10	0.0320
S18	0.0344	L10	0.0352	S20	0.0206	S16	0.0218	S18	0.0306
L24	0.0338	S20	0.0318	L24	0.0173	S10	0.0203	Thx	0.0299
S20	0.0332	L23	0.0285			Thx	0.0179	S16	0.0294
L23	0.0322	L29	0.0230			L31	0.0178	S20	0.0270
L29	0.0199	L24	0.0199			L29	0.0223	L29	0.0223
