# Supplementary material for: Network theory of the bacterial ribosome
Source: PLoS One. 2020 Oct 5;15(10):e0239700. doi: 10.1371/journal.pone.0239700 (PMC7535068; doi:10.1371/journal.pone.0239700)
Supplement: S7 Table — Note that the hubs used in Table 5 are shaded. (PDF) [file pone.0239700.s007.pdf]

S7 Table Closeness Centrality

decoding *(Thermus)*		decoding (*E. Coli)*		pre-peptide bond		mid-elongation		post-elongation	
**4v5g**	**value**	**5we4**	**value**	**4y4p**	**value**	**4v9h**	**value**	**4v9f**	**value**
23SrRNA-D2	0.5403	23SrRNA-D5	0.5194	tRNA-P	0.5289	23SrRNA-D5	0.5197	23SrRNA-D2	0.5447
23SrRNA-D5	0.5154	23SrRNA-D2	0.5194	23SrRNA-D2	0.5289	23SrRNA-D2	0.5197	EF-G	0.5276
tRNA-A	0.5076	tRNA-E	0.5115	tRNA-A	0.5161	EF-G	0.5038	23SrRNA-D5	0.5194
tRNA-P	0.4855	tRNA-P	0.4963	23SrRNA-D5	0.5079	tRNA-PE	0.4925	tRNA-P	0.4963
S13	0.4855	tRNA-A	0.4891	tRNA-E	0.5039	L14	0.4648	tRNA-E	0.482
tRNA-E	0.4855	mRNA	0.4558	23SrRNA-D0	0.4672	L35	0.449	S13	0.4653
L14	0.4653	L2	0.4558	L2	0.4571	L2	0.4459	L14	0.4621
L3	0.4558	16S-rRNA-CD	0.4527	L27	0.4539	L3	0.44	L2	0.4527
mRNA	0.4527	23SrRNA-D1	0.4497	S13	0.4507	L28	0.4314	L3	0.4437
L2	0.4527	L14	0.4408	mRNA	0.4507	S7	0.4314	L16	0.4437
S17	0.4437	S11	0.4408	L16	0.4507	L33	0.4314	mRNA	0.4437
23SrRNA-D4	0.4437	L16	0.4379	L14	0.4476	L6	0.4286	L35	0.4408
L35	0.4408	L3	0.4379	L35	0.4444	16S-rRNA-3'm	0.4258	S17	0.4408
L16	0.4379	16S-rRNA-3'M	0.4379	16S-rRNA-CD	0.4414	16S-rRNA-CD	0.4231	L27	0.4379
16S-rRNA-CD	0.4379	L35	0.4379	L3	0.4384	L32	0.4204	16S-rRNA-3'M	0.4379
L27	0.4379	5SrRNA	0.4351	23SrRNA-D4	0.4384	S19	0.4177	L6	0.4351
S11	0.4268	L27	0.4351	S17	0.4384	5SrRNA	0.4177	16S-rRNA-CD	0.4351
16S-rRNA-3'M	0.4268	S13	0.4295	16S-rRNA-3'M	0.4354	S11	0.4177	23SrRNA-D4	0.4295
L32	0.4268	23SrRNA-D4	0.4268	5SrRNA	0.4354	L13	0.4177	S11	0.4268
L33	0.4241	L28	0.4241	L28	0.4295	S15	0.4125	L32	0.4268
L13	0.4241	L33	0.4214	L32	0.4238	23SrRNA-D4	0.4099	L33	0.4241
L5	0.4188	L13	0.4188	L5	0.4238	L15	0.4099	L13	0.4214
L6	0.4188	L5	0.4188	L33	0.4238	S13	0.4099	5SrRNA	0.4214
5SrRNA	0.4188	16S-rRNA-3'm	0.4136	L13	0.4211	L1	0.4074	L5	0.4188
L19	0.4188	16S-rRNA-D5'	0.4136	L15	0.4183	L4	0.4074	L28	0.4188
16S-rRNA-3'm	0.4188	L6	0.4110	16S-rRNA-D5'	0.4156	16S-rRNA-3'M	0.4049	L15	0.4136
L28	0.4161	S15	0.4110	S15	0.4129	L19	0.4024	L11	0.4136
16S-rRNA-D5'	0.4161	L15	0.4085	L4	0.4103	L11	0.4	L4	0.411
L15	0.4136	S12	0.4061	16S-rRNA-3'm	0.4076	L16	0.3976	16S-rRNA-D5'	0.411
S15	0.4110	L4	0.4061	L34	0.4051	16S-rRNA-D5'	0.3976	S15	0.411
L4	0.4110	L19	0.3988	L20	0.4051	L5	0.3952	16S-rRNA-3'm	0.4085
L34	0.4085	L32	0.3988	L22	0.4000	23SrRNA-D6	0.3929	23SrRNA-D6	0.4061
S12	0.4012	S7	0.3988	L19	0.3975	L36	0.3929	L19	0.4012
L36	0.3988	L34	0.3964	S7	0.3951	L27	0.3929	S12	0.4012
23SrRNA-D1	0.3964	L20	0.3918	23SrRNA-D1	0.3926	mRNA	0.3929	L34	0.4012
L20	0.3941	L22	0.3918	L36	0.3902	S12	0.3929	L20	0.3988
L22	0.3941	L36	0.3895	L21	0.3902	L25	0.3905	L22	0.3964
EF-TU	0.3918	L21	0.3895	L6	0.3879	L20	0.3882	L36	0.3964
L1	0.3895	EF-TU	0.3873	23SrRNA-D3	0.3855	23SrRNA-D1	0.3882	L21	0.3941
S7	0.3829	L9	0.3873	S11	0.3832	L34	0.3882	L1	0.3895
L25	0.3807	S5	0.3807	S12	0.3765	L22	0.3882	S7	0.3873
L21	0.3807	S21	0.3807	S8	0.3765	L21	0.386	23SrRNA-D1	0.3851
S8	0.3785	S17	0.3764	L31	0.3765	23SrRNA-D0	0.3771	L25	0.3829
23SrRNA-D6	0.3743	23SrRNA-D6	0.3743	S9	0.3765	L30	0.3667	S8	0.3764
23SrRNA-D0	0.3722	L31	0.3743	L25	0.3743	S17	0.3646	L30	0.3743

decoding *(Thermus)*		decoding (*E. Coli)*		pre-peptide bond		mid-elongation		post-elongation	
**4v5g**	**value**	**5we4**	**value**	**4y4p**	**value**	**4v9h**	**value**	**4v9f**	**value**
L30	0.3722	L18	0.3722	L30	0.3743	L18	0.3646	S9	0.3743
S5	0.3702	L25	0.3722	L17	0.3721	23SrRNA-D3	0.3607	23SrRNA-D0	0.3722
L18	0.3681	23SrRNA-D0	0.3681	S5	0.3699	S5	0.3587	L18	0.3702
S6	0.3564	S6	0.3661	L9	0.3678	S9	0.3568	S5	0.3641
S3	0.3564	L30	0.3661	L18	0.3678	L17	0.3529	L10	0.3602
L11	0.3564	S9	0.3622	L23	0.3636	S2	0.3529	L17	0.3602
L31	0.3564	23SrRNA-D3	0.3602	23SrRNA-D6	0.3636	L10	0.3474	23SrRNA-D3	0.3583
23SrRNA-D3	0.3564	S3	0.3602	S6	0.3596	S18	0.3455	S6	0.3545
L10	0.3545	L11	0.3564	S3	0.3536	S14	0.3438	L12	0.3545
S9	0.3545	S8	0.3545	S2	0.3497	S3	0.3402	L31	0.3526
L17	0.3526	S18	0.3526	S19	0.3497	S8	0.3402	S19	0.3508
S4	0.3490	S4	0.3508	S4	0.3478	S6	0.3402	S3	0.349
Thx	0.3472	S2	0.3508	Thx	0.3368	L12	0.3402	S4	0.349
S19	0.3472	L17	0.3490	S16	0.3316	S4	0.335	S2	0.3436
S2	0.3384	L10	0.3454	S14	0.3232	S16	0.3267	Thx	0.3418
S16	0.3350	S19	0.3454	S10	0.3216	L23	0.3251	L24	0.335
L24	0.3350	S16	0.3418	S18	0.3184	L31	0.3204	S16	0.335
L23	0.3333	S14	0.3317	S20	0.3062	S20	0.3158	S18	0.3317
S18	0.3333	L23	0.3221	L29	0.2991	Thx	0.3084	L23	0.33
S20	0.3146	S10	0.3206	L24	0.2832	S10	0.3041	S14	0.319
S14	0.3131	L29	0.3160			L29	0.2845	S10	0.319
S10	0.3131	L24	0.3116			L24	0.2809	S20	0.3059
L29	0.2900	S20	0.3116					L29	0.2839
