# Supplementary material for: Network theory of the bacterial ribosome
Source: PLoS One. 2020 Oct 5;15(10):e0239700. doi: 10.1371/journal.pone.0239700 (PMC7535068; doi:10.1371/journal.pone.0239700)
Supplement: S8 Table — Note that the hubs used in Table 5 are shaded. (PDF) [file pone.0239700.s008.pdf]

S8 Table Betweenness Centrality

decoding *(Thermus)*		decoding (*E. Coli)*		pre-peptide bond		mid-elongation		post-elongation	
**4v5g**	**value**	**5we4**	**value**	**4y4p**	**value**	**4v9h**	**value**	**4v9f**	**value**
23SrRNA-D2	0.2666	23SrRNA-D2	0.2100	23SrRNA-D2	0.1923	23SrRNA-D2	0.2332	23SrRNA-D2	0.2515
23SrRNA-D5	0.1576	23SrRNA-D5	0.1665	23SrRNA-D5	0.1283	23SrRNA-D5	0.1993	23SrRNA-D5	0.1521
16S-rRNA-CD	0.0986	16S-rRNA-CD	0.1176	16S-rRNA-CD	0.1101	EF-G	0.1234	EF-G	0.1277
16S-rRNA-3'M	0.0828	23SrRNA-D1	0.1117	16S-rRNA-3'M	0.1012	tRNA-PE	0.1146	16S-rRNA-CD	0.0996
S13	0.0802	tRNA-E	0.1001	tRNA-P	0.0983	16S-rRNA-CD	0.1014	16S-rRNA-3'M	0.0991
tRNA-A	0.0667	16S-rRNA-3'M	0.0901	tRNA-E	0.0857	23SrRNA-D1	0.0816	tRNA-E	0.067
tRNA-P	0.0642	tRNA-P	0.0728	23SrRNA-D0	0.0797	16S-rRNA-D5	0.0684	tRNA-P	0.0643
tRNA-E	0.0636	tRNA-A	0.0657	tRNA-A	0.0758	16S-rRNA-3'M	0.0634	S13	0.0635
16S-rRNA-D5	0.0623	16S-rRNA-D5	0.0632	23SrRNA-D1	0.0709	16S-rRNA-3'm	0.0585	16S-rRNA-D5	0.0622
23SrRNA-D1	0.0599	L2	0.0463	16S-rRNA-D5	0.0638	S13	0.0446	23SrRNA-D1	0.0583
L3	0.0503	5SrRNA	0.0438	L2	0.0438	L14	0.0427	L2	0.0383
23SrRNA-D4	0.0406	L14	0.0351	S13	0.0428	S19	0.0408	L3	0.0372
L2	0.0394	16S-rRNA-3'm	0.0346	5SrRNA	0.0393	L2	0.0337	5SrRNA	0.0365
L14	0.0380	mRNA	0.0334	16S-rRNA-3'm	0.0341	S15	0.0335	L14	0.0354
mRNA	0.0346	S11	0.0268	S17	0.0316	5SrRNA	0.0295	23SrRNA-D4	0.0287
5SrRNA	0.0336	S13	0.0263	L14	0.0301	L28	0.0261	16S-rRNA-3'm	0.0278
16S-rRNA-3'm	0.0313	23SrRNA-D4	0.0246	23SrRNA-D4	0.0299	S7	0.0251	S17	0.0255
S17	0.0267	L3	0.0217	mRNA	0.0275	23SrRNA-D4	0.0242	S11	0.0215
S11	0.0213	23SrRNA-D6	0.0197	L28	0.0199	L35	0.021	L32	0.0206
L34	0.0211	S15	0.0167	L3	0.0164	L5	0.0204	23SrRNA-D6	0.0201
23SrRNA-D6	0.0172	L31	0.0154	23SrRNA-D3	0.0163	L34	0.0196	L34	0.0201
L32	0.0167	S5	0.0149	L35	0.0158	L3	0.0189	L28	0.0161
L28	0.0159	23SrRNA-D0	0.0147	S3	0.0146	23SrRNA-D6	0.0188	23SrRNA-D3	0.0157
23SrRNA-D3	0.0138	23SrRNA-D3	0.0145	L32	0.0145	L32	0.0186	23SrRNA-D0	0.0134
L19	0.0136	L13	0.0144	S5	0.0133	23SrRNA-D0	0.0161	L35	0.0132
S3	0.0133	L16	0.0139	L15	0.0131	S11	0.0161	mRNA	0.013
23SrRNA-D0	0.0125	L28	0.0134	L31	0.0127	L13	0.0146	L13	0.011
L35	0.0121	S3	0.0130	L23	0.0118	S5	0.0145	L19	0.0108
L13	0.0117	L32	0.0128	L13	0.0117	23SrRNA-D3	0.0145	S3	0.0107
S15	0.0107	EF-TU	0.0119	L19	0.0115	S3	0.0125	S9	0.0106
EF-TU	0.0103	L22	0.0113	L16	0.0108	L19	0.0115	S15	0.0103
S5	0.0085	L19	0.0104	L34	0.0107	L22	0.0086	S5	0.0097
L22	0.0082	S12	0.0095	23SrRNA-D6	0.0107	S9	0.0086	L11	0.0076
S12	0.0080	S21	0.0085	L27	0.0095	S12	0.0081	L22	0.0073
L6	0.0075	L6	0.0079	S15	0.0092	L15	0.008	L16	0.0069
L15	0.0071	L34	0.0075	S11	0.0082	L11	0.0078	S7	0.0065
L16	0.0070	L15	0.0061	S9	0.0080	L4	0.0065	L15	0.0065
L27	0.0064	S7	0.0061	S7	0.0075	S14	0.0061	L5	0.0063
S7	0.0063	L5	0.0054	L22	0.0051	S2	0.0042	L4	0.0052
S9	0.0061	L4	0.0051	L5	0.0051	L17	0.0038	L27	0.0052
L5	0.0056	L35	0.0050	L4	0.0051	L23	0.0036	S12	0.0048
S8	0.0051	L27	0.0049	L17	0.0049	S17	0.0035	S8	0.0045
L4	0.0049	L17	0.0048	L20	0.0046	mRNA	0.0033	L31	0.0043
L17	0.0043	S9	0.0047	S6	0.0045	L1	0.0032	L23	0.0038
L23	0.0037	S6	0.0046	L36	0.0031	S18	0.0031	L17	0.003

decoding *(Thermus)*		decoding (*E. Coli)*		pre-peptide bond		mid-elongation		post-elongation	
**4v5g**	**value**	**5we4**	**value**	**4y4p**	**value**	**4v9h**	**value**	**4v9f**	**value**
L20	0.0031	L20	0.0043	S8	0.0031	L20	0.0031	L10	0.0028
S6	0.0028	L9	0.0042	S2	0.0029	L21	0.0028	L20	0.0028
L36	0.0023	S17	0.0035	S19	0.0025	L36	0.0026	S6	0.0027
S2	0.0022	S14	0.0032	S12	0.0022	L6	0.0026	L21	0.0024
L33	0.0019	L21	0.0027	L6	0.0021	L33	0.0021	S2	0.0024
S4	0.0016	L36	0.0025	S4	0.0017	L27	0.002	S4	0.0023
L25	0.0013	L23	0.0018	L21	0.0014	S8	0.0016	L36	0.0022
L31	0.0011	L33	0.0012	S14	0.0014	L16	0.001	L12	0.002
L21	0.0010	S2	0.0010	L33	0.0013	S6	0.0008	S19	0.0019
S16	0.0005	S18	0.0009	L9	0.0007	S16	0.0005	L33	0.0016
L1	0.0003	S19	0.0009	S16	0.0004	S4	0.0005	L6	0.0015
S18	0.0003	S8	0.0008	S18	0.0004	L25	0.0003	L25	0.001
S10	0.0003	L11	0.0005	S10	0.0003	S10	0.0003	L24	0.0008
S14	0.0003	S16	0.0004	L30	0.0002	S20	0.0002	S16	0.0005
L11	0.0002	S4	0.0004	S20	0.0001	L24	0	S18	0.0003
S20	0.0001	S10	0.0002	L24	0.0000	L31	0	L1	0.0003
L24	0.0000	S20	0.0001	Thx	0.0000	Thx	0	S10	0.0003
S19	0.0000	L10	0.0000	L29	0.0000	L30	0	S14	0.0003
Thx	0.0000	L25	0.0000	L18	0.0000	L12	0	S20	0.0001
L18	0.0000	L24	0.0000	L25	0.0000	L29	0	Thx	0
L29	0.0000	L30	0.0000			L18	0	L18	0
L10	0.0000	L18	0.0000			L10	0	L30	0
L30	0.0000	L29	0.0000			L29	0.0000	L29	0
