# Supplementary figures and images for: Network theory of the bacterial ribosome
Source: PLoS One. 2020 Oct 5;15(10):e0239700. doi: 10.1371/journal.pone.0239700 (PMC7535068; doi:10.1371/journal.pone.0239700)

S1 Fig. Centrality measures for the pre-accommodated state of *T. Thermophilus* (pdb 4v5g)

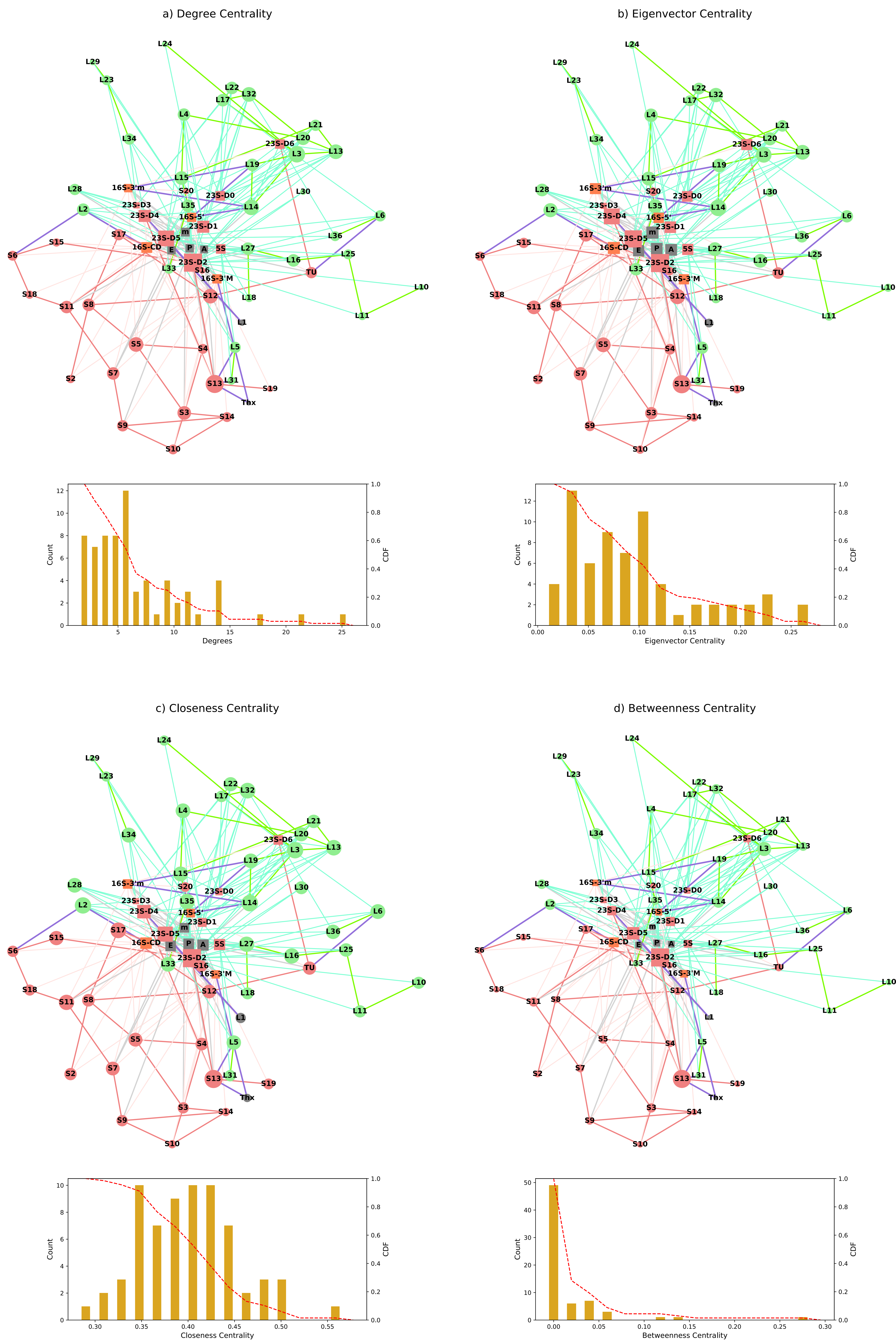

Supplement: S1 Fig — (PDF) [file pone.0239700.s009.pdf]

S2 Fig. Centrality measures for the accommodated state of *T. Thermophilus* (pdb 4y4p)

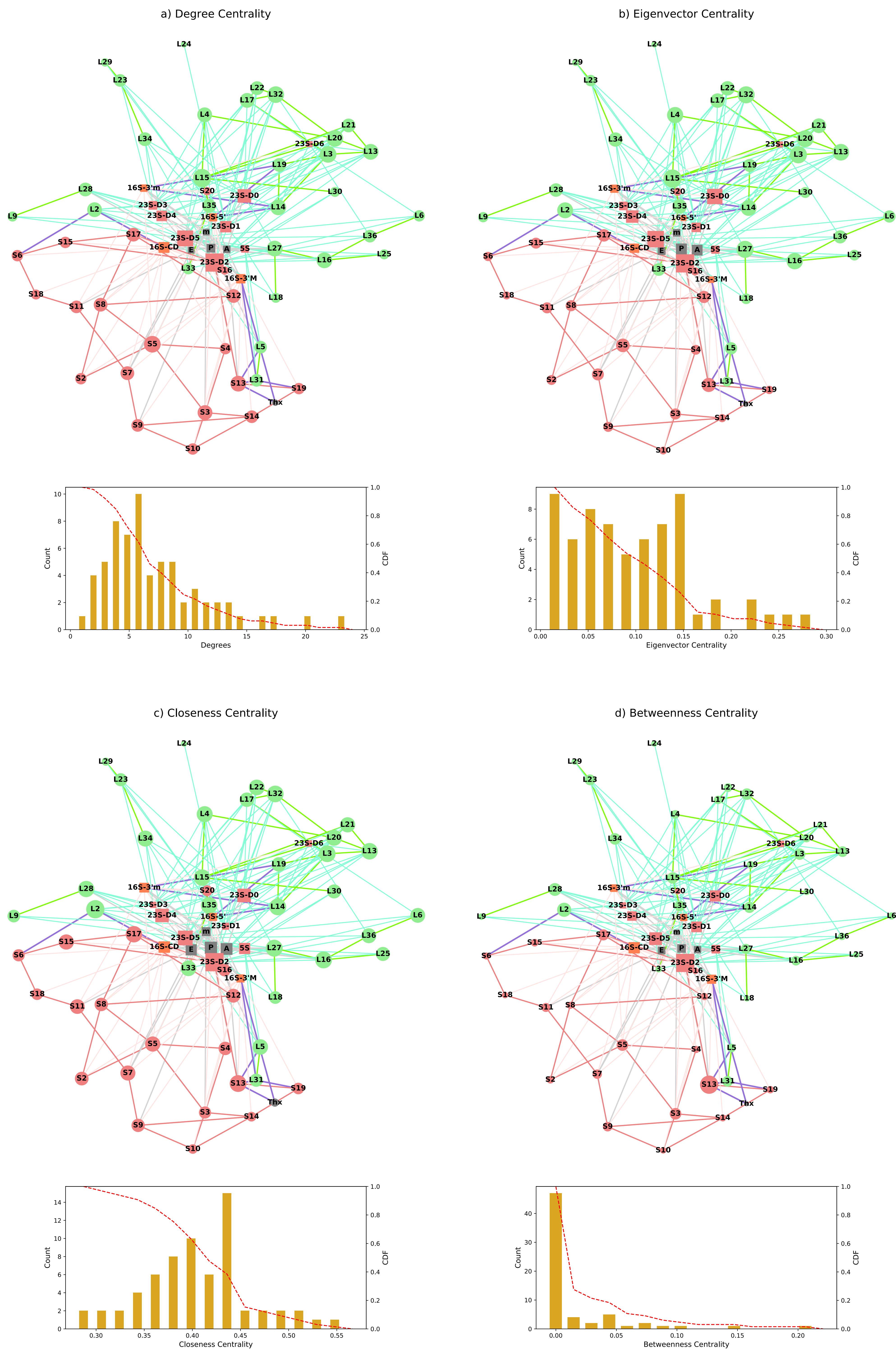

Supplement: S2 Fig — (PDF) [file pone.0239700.s010.pdf]

S3 Fig. Centrality measures for the hybrid state of *T. Thermophilus* (pdb 4v9h)

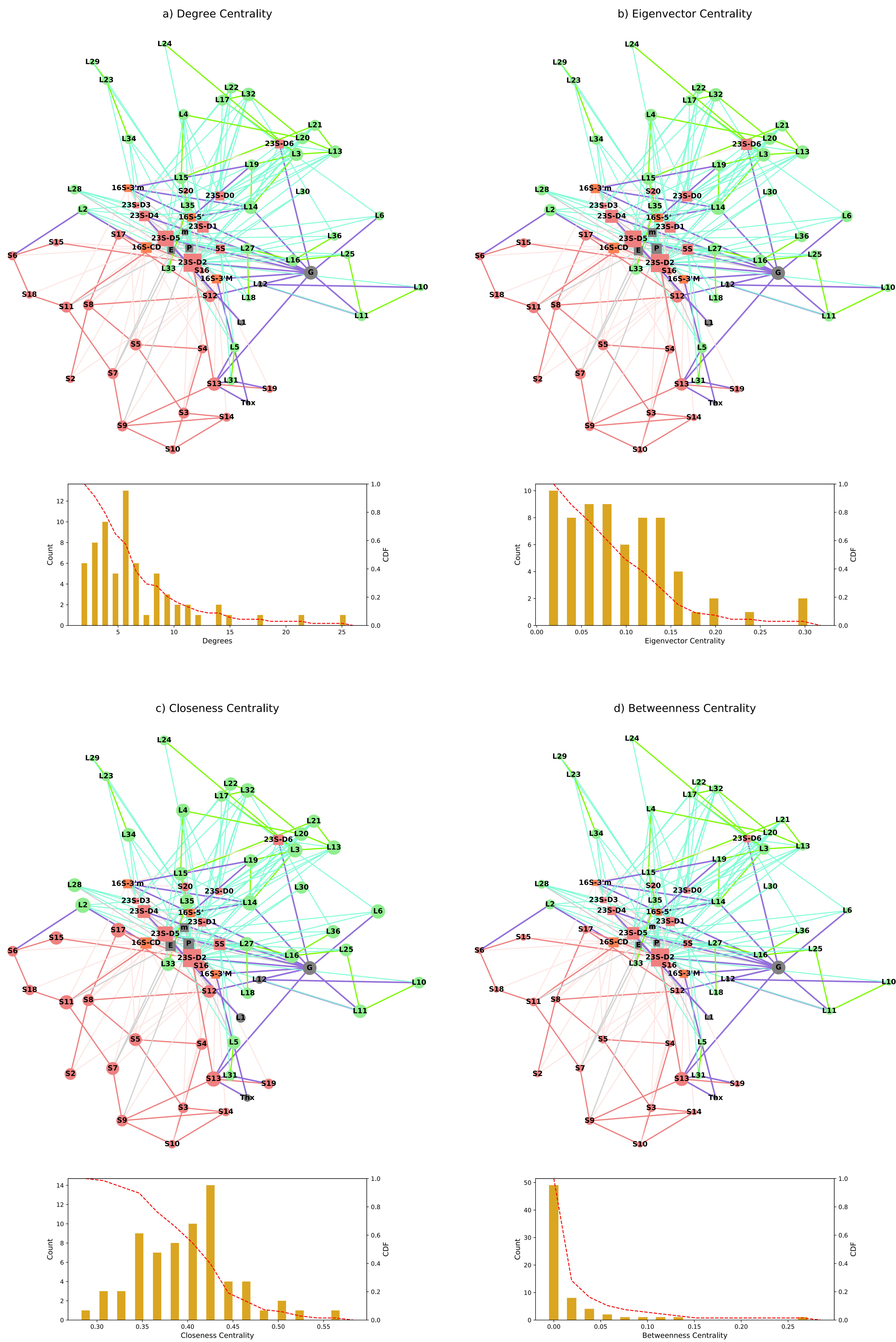

Supplement: S3 Fig — (PDF) [file pone.0239700.s011.pdf]

S4 Fig. Centrality measures for the pre-accommodated state of *T. Thermophilus* (pdb 4v5f)

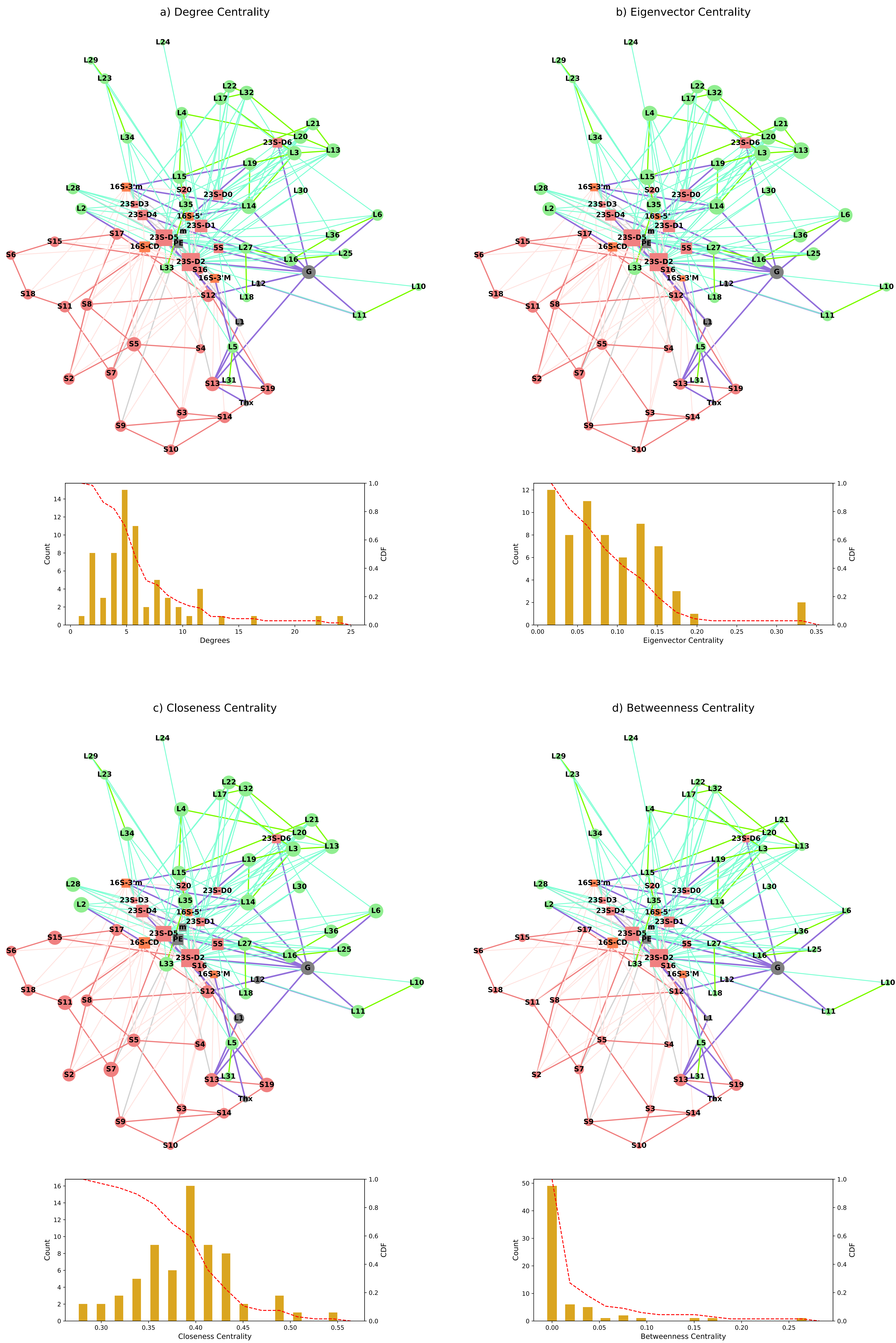

Supplement: S4 Fig — (PDF) [file pone.0239700.s012.pdf]
